# Extracellular Metabolite Profiling in CO_2_-Fixing Bacterium *Rhodobacter sphaeroides* Under Autotrophic Conditions

**DOI:** 10.3390/metabo16030156

**Published:** 2026-02-26

**Authors:** Yu Rim Lee, Suhyeon Hong, Young-Hwan Chu, Soo Youn Lee, Sangmin Lee

**Affiliations:** 1 Gwangju Clean Energy Research Center, Korea Institute of Energy Research, Gwangju 61003, Republic of Korea; rimin627@gmail.com; 2 Department of Agricultural Chemistry, Chungnam National University, Daejeon 34134, Republic of Korea; shhong@o.cnu.ac.kr; 3 Energy AI & Computational Science Laboratory, Korea Institute of Energy Research, Daejeon 34129, Republic of Korea; yhchu@kier.re.kr; 4 Department of Bio-Environmental Chemistry, Chungnam National University, Daejeon 34134, Republic of Korea

**Keywords:** *Rhodobacter sphaeroides*, autotrophic culture, CE-TOFMS, LC-TOFMS, metabolomics

## Abstract

**Background/Objectives:** *Rhodobacter sphaeriids* is considered a promising biomanufacturing platform due to its capacity to convert CO_2_ into value-added products. To enhance the yield of CO_2_-derived products, understanding extracellular metabolite dynamics during autotrophic growth is essential. However, the extracellular metabolite profiles of *R. sphaeroides* under autotrophic conditions have not been reported. **Methods:** In this study, we performed a comprehensive analysis of extracellular metabolites produced under autotrophic conditions using capillary electrophoresis time-of-flight mass spectrometry (CE-TOFMS) and liquid chromatography time-of-flight mass spectrometry (LC-TOFMS). **Results:** A total of 62 putative metabolites were detected, of which 23 were measured above the quantification limit. Metabolites involved in glycolysis and gluconeogenesis constituted the largest proportion of extracellular metabolites, with lactic acid exhibiting the highest accumulation levels. To investigate the transcriptional changes associated with metabolite accumulation, we analyzed gene expression and observed the downregulation of glycolytic genes, including *pgi*, *gapB*, and *lctB*, whereas *cfxA*, encoding fructose-1,6-bisphosphate aldolase, was upregulated under autotrophic conditions compared to heterotrophic conditions. **Conclusions:** These results suggest that the carbon assimilation metabolic flux in *R. sphaeroides* shifts toward the CBB cycle and lactic acid overflow metabolism under autotrophic conditions. Collectively, these findings provide new insights into metabolic regulation during autotrophic growth and offer a basis for reducing extracellular byproduct formation and improving CO_2_-based biological production in *R. sphaeroides*.

## 1. Introduction

Global CO_2_ emissions reached 35.8 GtCO_2_ in 2023, further eroding the remaining carbon budget for a 1.5 °C warming limit, which is projected to be depleted within 0.5–6 years, thereby underscoring the urgent need for a paradigm shift toward a carbon-circular economy [1]. Although biological CO_2_ conversion offers substantial promise for producing value-added chemicals and fuels, it faces significant scalability challenges compared to physicochemical carbon capture and utilization technologies as no single approach currently delivers a comprehensive large-scale solution [2,3,4].

This transition is pivotal for mitigating climate change and cultivating emerging bio-industries [5]. Worldwide initiatives to curb greenhouse gas emissions increasingly emphasize biological CO_2_ conversion, which uniquely enables simultaneous atmospheric CO_2_ reduction and synthesis of high-value products [6].

*Rhodobacter sphaeroides* is a versatile α-proteobacterium that has gained significant attention as a novel microbial cell factory due to its ability to synthesize compounds vital for the bioeconomy, including isoprenoids, poly-β-hydroxybutyrate, and hydrogen [7]. This organism offers several advantages, including non-pathogenicity, a wide range of utilizable substrates, useful metabolic pathways, and amenability to genetic modification for the production of high-value chemicals [8]. As a photosynthetic bacterium, *R. sphaeroides* exhibits broad metabolic versatility, enabling the utilization of various carbon sources, including CO_2_ via the Calvin–Benson–Bassham pathway, in addition to sugars and organic acids [6].

Beyond its non-pathogenicity and broad substrate utilization, *R. sphaeroides* stands out from other microbial cell factories like *Escherichia coli* or *Saccharomyces cerevisiae* due to its unique metabolic flexibility, including both photoautotrophic and chemoheterotrophic growth capabilities [7]. This inherent metabolic versatility allows it to efficiently fix CO_2_ through the Calvin–Benson–Bassham pathway and channel carbon into diverse biosynthetic routes. Previous studies have successfully leveraged *R. sphaeroides* for the sustainable production of critical industrial precursors and biofuels, such as isoprene, poly-β-hydroxybutyrate as a biopolymer, and even hydrogen as a clean energy carrier, demonstrating its robust potential as a chassis for sustainable biomanufacturing [7,9,10,11].

Following carbon fixation, terpenoids such as isoprene are synthesized through the methylerythritol 4-phosphate pathway. In this pathway, glyceraldehyde-3-phosphate and pyruvate are converted into basic C5 precursors, such as isopentenyl pyrophosphate (IPP) and dimethylallyl pyrophosphate (DMAPP), through a series of reactions. Geranyl pyrophosphate (GPP) and farnesyl pyrophosphate (FPP) are then synthesized and directly used for the biosynthesis of terpenoids [8,12].

Prior studies have established the versatility of *R. sphaeroides* in synthesizing diverse compounds from varied carbon sources. Investigations have encompassed metabolites such as isoprene and lipophilic biofuels, alongside metabolic engineering approaches for producing β-farnesene, a precursor to bio-jet fuel [13]. Herein, we demonstrate the metabolic engineering of *R. sphaeroides* to produce β-farnesene from CO_2_ under photoautotrophic conditions. Heterologous expression of a codon-optimized farnesene synthase from *Citrus junos* enabled the direct conversion of CO_2_ into this sesquiterpene precursor via the methylerythritol 4-phosphate pathway [13]. Optimization of culture conditions, including supplementation with additional media components and ascorbic acid, substantially elevated β-farnesene titers. Ultimately, β-farnesene was produced from CO_2_ at a titer of 44.53 mg/L and a yield of 234.08 mg/g CO_2_.

To enhance product titers, elucidating carbon flux distribution and implementing metabolic engineering strategies to eliminate competing pathways are essential. Comprehensive metabolomic profiling under autotrophic conditions is therefore crucial. Furthermore, the discovery of novel metabolites is feasible.

To acquire a holistic understanding of cellular physiology and optimize microbial strains, both metabolomics and transcriptomics play indispensable roles [14]. Metabolomics provides a snapshot of the cellular metabolic state, directly reflecting enzyme activities and pathway fluxes, while transcriptomics reveals the gene expression patterns that govern enzyme synthesis. However, a ‘single-omics’ approach often yields an incomplete picture as post-transcriptional, translational, and post-translational regulatory mechanisms can lead to significant discrepancies between mRNA levels and corresponding metabolite concentrations [15]. Therefore, an integrated multi-omics analysis, combining these powerful tools, is critical to unravel complex regulatory networks, identify key metabolic bottlenecks, and ultimately accelerate the rational design of microbial cell factories for enhanced production of target compounds [16].

Among the detected metabolites, lactic acid was observed at notably higher concentrations than other metabolites. Lactic acid is a key precursor for various materials like bioplastics, food additives, and pharmaceutical compounds [11]. It is a valuable platform chemical with diverse industrial applications, and its substantial accumulation under autotrophic conditions suggests a significant metabolic flux toward its production in *R. sphaeroides*. For instance, metabolic engineering of *Rhodococcus opacus* DSM 43205 has enabled the production of L-lactic acid as a biopolymer precursor directly from CO_2_ under autotrophic conditions [17]. This demonstrates the feasibility of microbial lactic acid bioproduction from CO_2_, paralleling the high lactic acid accumulation observed in *R. sphaeroides* and indicating potential for metabolic engineering strategies to either redirect carbon flux toward target products or to valorize lactic acid itself through subsequent conversions.

In this study, water-soluble metabolites were analyzed using capillary electrophoresis time-of-flight mass spectrometry (CE-TOFMS), while fat-soluble metabolites, such as fatty acids, were analyzed using liquid chromatography time-of-flight mass spectrometry (LC-TOFMS). We performed comprehensive metabolomic profiling of *R. sphaeroides* under autotrophic culture conditions coupled with RNA sequencing to compare the gene expression patterns of relevant enzymes under autotrophic and heterotrophic conditions. The resulting data will aid the identification of industrially useful substances, reveal the potential utility of *R. sphaeroides*, and assist efforts to reduce byproducts while enhancing metabolic flux toward high-value chemical production.

## 2. Materials and Methods

### 2.1. Bacterial Growth Conditions and Preparation of Analysis Samples

An *R. sphaeroides*-type strain (KCTC 1434) was obtained from the Korean Collection for Type Cultures (KCTC). *R. sphaeroides* was grown in modified Sistrom’s minimal medium as previously described [18]. The modified Sistrom’s medium consisted of the following components (L^−1^): 2.72 g of KH_2_PO_4_, 1.95 g of NH_4_Cl, 4 g of succinic acid, 0.1 g of glutamic acid, 40 mg of aspartic acid, 0.5 g of NaCl, 0.2 g of nitriloacetic acid, 0.3 g of MgSO_4_·7H_2_O, 33.4 mg of CaCl_2_·2H_2_O, 2 mg of FeSO_4_·7H_2_O, 2 mg of (NH_4_)_6_Mo_7_O_24_, 100 microliters of a trace elements solution, and 100 µL of a vitamins solution. The trace element solution was formulated with the following components (100 mL^−1^): 1.765 g of EDTA, 10.95 g of ZnSO_4_·7H_2_O, 5.0 g of FeSO_4_·7H_2_O, 1.54 g of MnSO_4_·H_2_O, 0.392 g of CuSO_4_·5H_2_O, 0.284 g of Co(NO_3_)·6H_2_O, and 0.11 g of H_3_BO_3_. The vitamin solution was formulated with the following composition (100 mL^−1^): 1 g of nicotinic acid, 0.5 g of thiamin HCl, and 0.1 g of biotin. The medium was adjusted to pH 7.0 using a 20% (*w*/*v*) KOH solution.

Pre-culture cells were prepared by inoculating cells at 1% (*v*/*v*) into 20 mL of Sistrom’s medium in a 50mL conical tube and incubated for 48 h. For autotrophic cultivation, the pre-cultured cells were inoculated into 100 mL of modified growth medium in serum bottles after being diluted to OD660 of 0.1. To maintain autotrophic conditions, cultures were purged with an oxygen-free gas mixture containing CO_2_ (5%), H_2_ (60%), and Ar (35%). CO_2_ was supplied as the sole carbon source instead of succinic acid. For heterotrophic cultivation, pre-cultured cells were diluted to an OD660 of 0.1 and inoculated into 100 mL of Sistrom’s growth medium containing succinic acid in a 250 mL Erlenmeyer flask. The cells were cultivated at 30 °C with shaking at 150 rpm under continuous light conditions for 48 h (heterotrophic conditions) and 14 days (autotrophic conditions). Light was supplied using white LED lamps (450–475 nm) at 28 ± 3 °C, providing a light intensity of 50 µmol photons m^−2^s^−1^. Cell growth was monitored by measuring optical density (OD) using a spectrophotometer (BioSpectrometer, Eppendorf, Hamburg, Germany) at 660 nm. All cultivation experiments were performed in triplicate.

**Figure 1:**
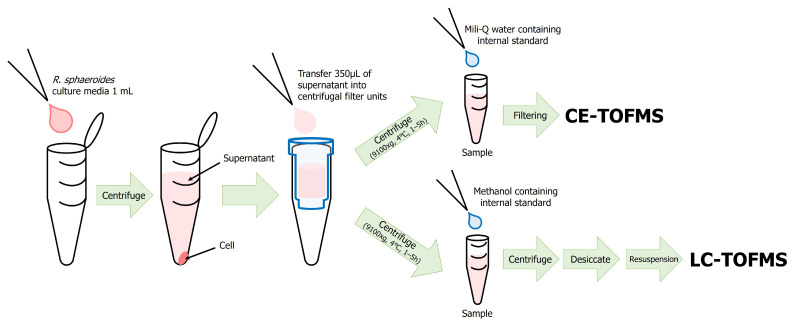
Sample preparation procedure for extracellular metabolite extraction.

To prepare samples for metabolomic analysis, the culture broth of *R. sphaeroides* was centrifuged, and only the supernatant was collected [19,20] (Figure 1). Samples were harvested from the cultures at the end of the cultivation period, corresponding to day 14 for autotrophic conditions. For CE-TOFMS analysis, 80 µL of the supernatant was mixed with 20 µL of Milli-Q water containing internal standards, trimesic acid and 3-hydroxynaphthalene-2,7-disulfonic acid to calibrate the retention time for CE. To remove macromolecules, the mixtures were filtered using a 5 kDa cut-off filter (ULTRAFREE-MC-PLHCC, Human Metabolome Technologies, Yamagata, Japan). For LC-TOFMS analysis, 200 µL of the supernatant was mixed with 600 µL of methanol containing internal standards, namely, methionine sulfone and D-camphor-10-sulfonic acid (CSA), to calibrate the retention time for LC, and centrifuged at 2300× *g* for 5 min at 4 °C. The resulting supernatant was dried and immediately resuspended in 200 µL of an isopropanol–water mixture (1:1, *v*/*v*) prior to analysis.

### 2.2. Metabolites Identification and Quantification

Metabolite identification and quantification in *R. sphaeroides* were performed using CE-TOFMS and LC-TOFMS by Human Metabolome Technologies (Yamagata, Japan). Hydrophilic and highly polar metabolites were analyzed using the positive ion and negative ion modes of CE-TOFMS (Agilent 7100 CE-TOFMS system, Santa Clara, CA, USA) equipped with a fused silica capillary (80 cm, 50 µm ID) [21]. Sample injection was conducted at 50 mbar for 10 s. The applied CE voltage and MS capillary voltage were +30 kV and +4 kV, respectively, and the MS scan range was set from *m*/*z* 50–1000. Lipophilic and relative non-polar metabolites were analyzed using LC-TOFMS (Agilent 1200 RRLC system SL, Santa Clara, CA, USA) in positive and negative modes with an ODS column (2 × 50 mm, 2 µm ID) [22]. The column temperature was maintained at 40 °C. Mobile phases consisted of water containing 0.1% formic acid and an isopropanol–acetonitrile–water mixture (65:30:5, *v*/*v*) containing 0.1% formic acid and 2 mM ammonium formate. The flow rate was 0.3 mL/min. The MS gas flow rate was 10 L/min, with a gas temperature of 350 °C, and the MS scan range was *m*/*z* 100–1700.

Data processing was performed using automatic integration software to obtain peak information, including *m*/*z* values, migration time for CE, retention time for LC, and peak area. Peak areas were converted to relative peak areas using the following equation:

$$\mathrm{Relative}\;\mathrm{Peak}\;\mathrm{Area}\;=\;\frac{Metabolite\;Peak\;Area}{Internal\;Standard\;Peak\;Area\times Sample\;Amount}$$

The peak detection limit was determined based on a signal-to-noise (S/N) ratio of 3.

Putative metabolites were assigned from HMT’s standard library and Known-Unknown peak library based on *m*/*z*, MT, or tR values, with tolerances of ±0.5 min, ±0.3 min, ±10 ppm, and ±25 ppm (*m*/*z*). If multiple peaks matched the same candidate, a branch number was assigned to the candidate. The limit of detection (LoD) was determined using background signal levels from blank samples (modified Sistrom’s medium without *R. sphaeroides* cultivation) and signal-to-noise ratio criteria (S/N ≥ 3).

Absolute quantification was performed for target metabolites by normalizing peak areas to internal standards and applying standard curves generated using single-point calibration at 100 µM.

### 2.3. Gene Expression Profiling

Total RNA samples for transcriptome analysis were extracted from the harvested cells at the end of the cultivation period, corresponding to day 14 for autotrophic conditions and 48 h for heterotrophic conditions. Harvested cells were washed twice with DNase/RNase-free distilled water, and total RNA was isolated according to the manufacturer’s protocol using the Quick-RNA Fungal/Bacterial Miniprep Kit (Zymo Research, Irvine, CA, USA). Genomic DNA contamination was removed by treatment with RNase-free DNase I. Extracted RNA samples were quantified using a NanoDrop 2000 spectrophotometer (Thermo Fischer Scientific, Waltham, MA, USA). Complementary DNA (cDNA) library construction and the initial processing of raw sequencing data were performed by Macrogen (Seoul, Republic of Korea). The resulting cDNA libraries were then sequenced on an Illumina HiSeq 2500 platform (llumina, Inc., San Diego, CA, USA) in paired-end mode. Quality control of raw sequencing reads was conducted to assess overall read quality, including total reads, total bases, and GC content. To minimize analytical bias, pre-processing steps were conducted to remove low-quality reads and technical artifacts, including adaptor sequences, contaminant DNA, and PCR duplicates. After that, filtered reads were mapped to the reference genome using the Bowtie alignment program, and aligned reads were generated. Gene-level read counts were calculated from the aligned reads mapped to annotated gene regions. Gene expression profiles for each sample were obtained by normalizing read counts according to transcript length and sequencing depth, and expression levels were calculated as FPKM (Fragments Per Kilobase of transcript per Million mapped reads) or RPKM (Reads Per Kilobase of transcript per Million mapped reads). Differential gene expression analysis was conducted using the edgeR package, and genes were considered differentially expressed based on a *p*-value < 0.05 and a fold change > 2. The reference genome of *R. sphaeroides* for transcriptome analysis was obtained from the NCBI database (accession number: GCF_003324715.1, access date: 15 July 2024).

## 3. Results

### 3.1. Extracellular Metabolite Profiles of R. sphaeroides Under Autotrophic Conditions

To maximize the efficiency of bioprocesses for the production of high-value chemicals, it is necessary to identify the metabolic byproducts that are released into the extracellular environment during microbial cultivation. Reducing extracellular carbon loss enables more effective substrate utilization and increases the productivity of target products [23]. Despite the growing interest in *R. sphaeroides* as a sustainable biomanufacturing platform, comprehensive extracellular metabolite profiling under autotrophic conditions, in which CO_2_ serves as the sole carbon source, has not been reported.

To address this gap, extracellular metabolites produced by *R. sphaeroides* during autotrophic growth were analyzed using CE-TOFMS and LC-TOFMS. CE-TOFMS was primarily used for the detection of hydrophilic and highly polar metabolites, while LC-TOFMS was mainly utilized for the detection of lipophilic and relatively non-polar metabolites. To remove background signals and subtract contaminations, modified Sistrom’s medium without *R. sphaeroides* was used as a blank sample. In total, 78 putative metabolite peaks were identified and annotated using HMT’s standard library and Known-Unknown peak library (Table 1). Among these, 61 metabolites were identified using CE-TOFMS, including 28 metabolites detected in positive ion mode and 33 metabolites detected in negative ion mode. Additionally, 17 metabolites were identified using LC-TOFMS, including 9 metabolites detected in positive ionization mode and 8 metabolites detected in negative ionization mode.

Among the 78 annotated putative metabolite peaks, relative peak areas were quantified for 62 putative metabolites; 13 peaks were below the limit of detection (LoD), and the remaining 3 were analytical artifacts.

Among the metabolites, methyl sulfate, N-Ethylmaleimide+H_2_O, and phenol are analytical artifacts rather than genuine products of *R. sphaeroides*. Methyl sulfate arises from methanol used in sample preparation. This phenomenon is well-documented in metabolomics research, where using methanol steps from sample collection to instrumental analysis can introduce non-biological compounds or alter endogenous metabolites [24]. N-Ethylmaleimide+H_2_O results from the thiol-protecting reagent applied during processing [25]. Furthermore, phenol is also considered an artifact. Its appearance could be attributed to contamination or chemical reactions occurring during the extensive sample extraction and analysis procedures [24]. Therefore, all three compounds, methyl sulfate, N-Ethylmaleimide+H_2_O, and phenol, are interpreted as analytical artifacts rather than metabolic products of *R. sphaeroides*.

Next, absolute quantification was performed for detected metabolites using internal-standard normalization and single-point calibration. Among the 62 metabolites identified by CE-TOFMS and LC-TOFMS, 23 metabolites were detected at levels above the limit of quantification under autotrophic conditions (Table 2). All 23 quantified metabolites were detected by CE-TOFMS, indicating that metabolites produced by *R. sphaeroides* at quantifiable levels under autotrophic conditions were mainly hydrophilic and polar compounds. Based on their associated metabolic pathways, the quantified metabolites were classified into three major groups: (i) glycolysis- and gluconeogenesis-related metabolites; (ii) TCA cycle-related metabolites; and (iii) metabolites associated with glutamate metabolism and the urea cycle.

Glycolysis- and gluconeogenesis-related metabolites constituted the largest group within the overall extracellular metabolite profile. Among these, lactic acid exhibited the highest accumulation at 4.9 µM. Glycolysis intermediates, including 3-phosphoglyceric acid, glucose-6-phosphate, and phosphoenolpyruvic acid, were detected at concentrations of 1.7 µM, 0.5 µM, and 1.6 µM. In addition, amino acids branching from glycolytic intermediates, such as glycine, serine, and threonine, were identified. These results suggest that lactic acid serves as a major carbon overflow metabolite under autotrophic conditions. Furthermore, the presence of glycolysis-related intermediates in the extracellular space indicates that a portion of CO_2_-derived fixed carbon is not fully processed through central carbon metabolism and is instead partially released from the cell.

Metabolites associated with the TCA cycle represented the second largest group among the quantified compounds. 3-Hydroxybutryic acid, a metabolite involved in redox-balancing reactions, accumulated at 0.9 µM. Several amino acid-derived TCA cycle-related intermediates, including alanine, isoleucine, leucine, and lysine, were detected at low micromolar concentrations. Glutamic acid and glutamine, both closely linked to α-ketoglutarate, were each detected at 0.8 µM. These findings indicate that TCA cycle-associated metabolites are also released into the extracellular environment during autotrophic growth.

A third group of metabolites was associated with glutamate metabolism and the urea cycle. Creatine and histidine were detected at approximately 0.2 µM, while proline and ornithine accumulated at 0.4 µM. S-adenosylmethionine (SAM) was detected at 0.4 µM, and the polyamine spermidine was detected at 0.08 µM. Moreover, glyoxylic acid and gluconic acid, which are associated with choline metabolism, the methionine salvage pathway, and the gluconate shunt pathway, were quantified at 2.8 µM and 0.7 µM, respectively. The release of metabolites related to glutamate metabolism and the urea cycle may reflect byproducts of intracellular amino acid turnover or nitrogen redistribution processes involved in maintaining cellular nitrogen balance [25].

### 3.2. Transcript Levels Related to Central Carbon Metabolism in R. sphaeroides Under Autotrophic Conditions

Metabolites represent the end products of cellular metabolism and reflect the integrated outcomes of genetic and environmental influences. Their accumulation is influenced by enzyme activities related to gene expression [26]. In this study, glycolysis- and gluconeogenesis-related metabolites constituted the largest proportion of detected metabolites, with lactic acid showing the highest extracellular concentration. Accordingly, to explore transcriptional alterations associated with the accumulation of metabolites involved in central carbon metabolism, we analyzed the transcript levels of *R. sphaeroides* under autotrophic conditions. To minimize sample variability between transcriptomic and metabolomics analyses, samples were collected at the same cultivation endpoint. Heterotrophic cultures were also used as a control condition in transcriptomic analysis to assess transcriptional changes under autotrophic condition relative to general cultivation conditions. According to transcriptomic analysis, a total of 1004 genes were identified and showed significant differential expression between autotrophic and heterotrophic conditions (Table S1). Among these differentially expressed genes, gene ontology (GO) analysis revealed that most genes were assigned to the molecular function (36.82%) and biological process (36.22%), followed by the cellular component (10.33%), while 16.64% showed no GO annotation (Figure S1).

**Figure 2:**
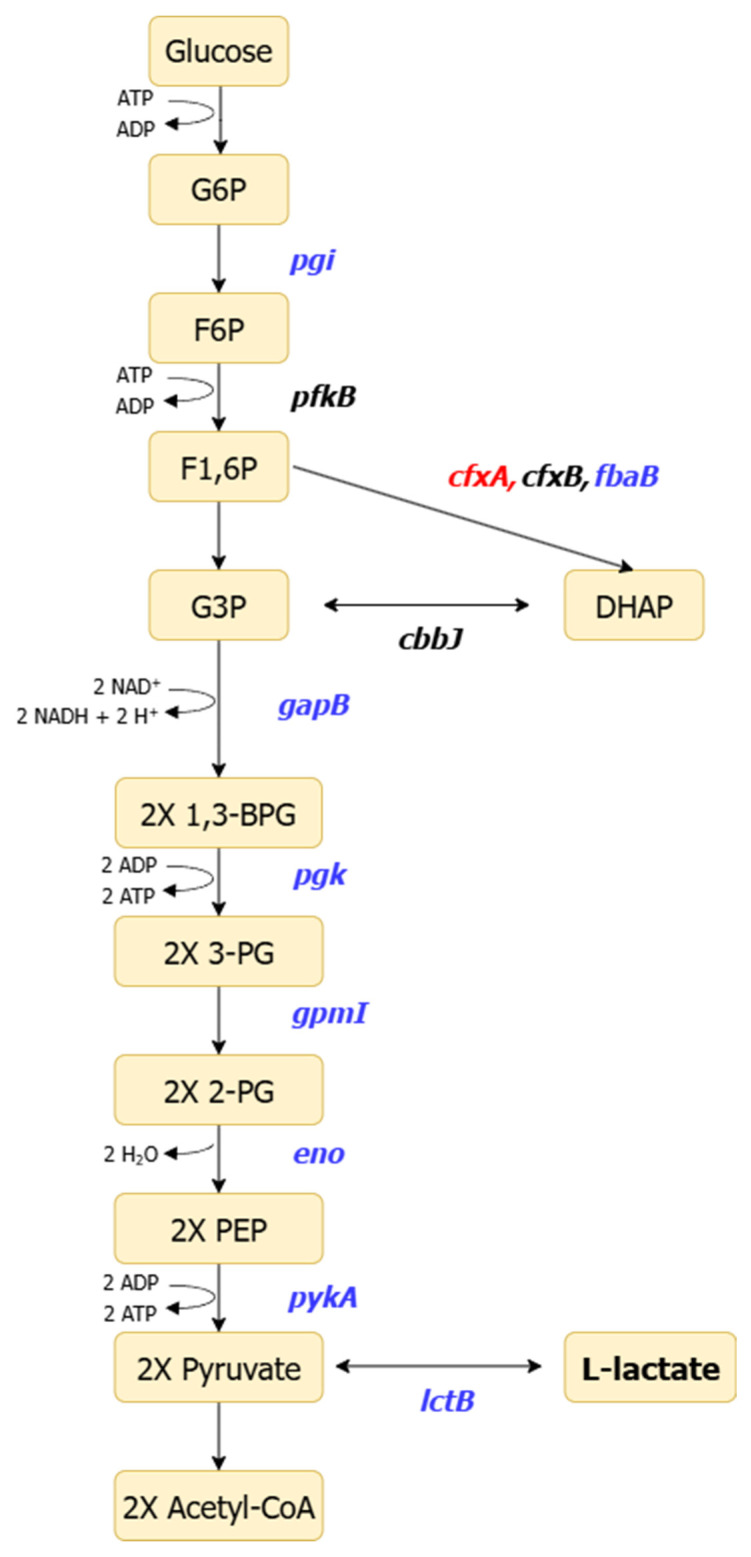
Schematic representation of the glycolytic pathway and relative transcript expression of associated genes in *R. sphaeroides* under autotrophic conditions. Genes with decreased transcript expression are shown in blue italics, genes with increased expression are shown in red italics, and genes not detected in the transcriptome analysis are shown in black italics. The abbreviations of enzymes are as follows: *pgi*, phosphoglucose isomerase; *pfkB*, phosphofructokinase B; *cfxA*, fructose-bisphosphate aldolase A; *cfxB*, fructose-bisphosphate aldolase B; *fbaB*, fructose-bisphosphate aldolase B; *gapB*, glyceraldehyde-3-phosphate dehydrogenase B; *pgk*, phosphoglycerate kinase; *gpmI*, phosphoglycerate mutase I; *eno,* enolase; *pykA*, pyruvate kinase A; *lctB*, lactate dehydrogenase B. Pathway intermediates are abbreviated as follows: G6P, glucose-6-phosphate; F6P, fructose-6-phosphate; F1,6P, fructose-1,6-bisphosphate; G3P, glyceraldehyde-3-phosphate; DHAP, dihydroxyacetone phosphate; 1,3-BPG, 1,3-bisphosphoglycerate; 3-PG, 3-phosphoglycerate; 2-PG, 2-phosphoglycerate; PEP, phosphoenolpyruvate. Arrows indicate the direction of metabolic flux between intermediates.

In addition, transcriptomic analysis results focusing on central carbon metabolism showed that genes encoding key enzymes of the glycolytic pathway, such as *pgi*, *fbaB*, *gapB*, *pgk*, *gpmI*, *eno*, and *pykA*, were broadly downregulated under autotrophic conditions compared to heterotrophic conditions, in which succinic acid is utilized as the carbon source (Figure 2 and Table 3) [27]. The transcript expression levels of glyceraldehyde-3-phosphate dehydrogenase, encoded by *gapB*, and enolase, encoded by *eno*, were decreased by more than two-fold under autotrophic conditions. Transcript levels of the lactate dehydrogenase gene *lctB* were also reduced under autotrophic conditions compared to heterotrophic conditions. In contrast, the *cfxA* gene, encoding fructose-1,6-bisphosphate aldolase A, was markedly upregulated under autotrophic conditions.

Previous studies have reported that genes associated with the Calvin–Benson–Bassham (CBB) cycle are upregulated to support CO_2_ assimilation, whereas genes involved in the TCA cycle and organic carbon metabolism are downregulated during autotrophic conditions [19]. When microorganisms utilize CO_2_ as the sole carbon source, carbon metabolism is closely linked to CO_2_ fixation, resulting in a metabolic shift in which the CBB cycle serves as the primary carbon assimilation pathway [27]. *R. sphaeroides* modulates the CBB cycle through two *cbb* operons, *cbb_I_* and *cbb_II_*, which enable the fixation of carbon dioxide and its utilization as a carbon source under photoautotrophic and chemoautotrophic conditions [28]. Similarly, many cyanobacteria and purple non-sulfur bacteria, including *Rhodospirillum rubrum* and *Cupriavidus necator* (formerly known as *Ralstonia eutropha*), also possess the CBB cycle and are capable of assimilating carbon dioxide for their cellular growth and metabolite production [29,30,31,32]. Under these conditions, glycolysis functions mainly as a downstream carbon-processing pathway following the CBB cycle [27]. Accordingly, the observed downregulation of glycolysis-related genes in this study likely reflects metabolic reorganization under autotrophic conditions.

In addition, fructose-1,6-bisphosphate aldolase plays a dual role in cellular metabolism. In glycolysis, it catalyzes the cleavage of fructose-1,6-bisphosphate (F1,6P) into glyceraldehyde-3-phosphate (G3P) and dihydroxyacetone phosphate (DHAP), enabling carbon flux into downstream reactions involved in Adenosine Triphosphate (ATP) and Nicotinamide adenine dinucleotide (NADH) production (Figure 2). In contrast, within the CBB cycle, this enzyme catalyzes the reverse reaction and contributes to ribulose-1,5-bisphosphate (RuBP) regeneration through sugar-phosphate rearrangements. This functional duality may account for the increased transcript level of *cfxA* observed under autotrophic conditions. Altogether, these results indicate that autotrophic growth in *R. sphaeroides* drives a metabolic shift toward CBB cycle-centered carbon assimilation, accompanied by reduced expression of glycolytic genes.

## 4. Discussion

Extracellular metabolites produced by *R. sphaeroides* under autotrophic conditions were identified through metabolomics analysis in this study. A total of 23 extracellular metabolites were quantified under autotrophic conditions, and these metabolites were categorized into three groups related to glycolysis and gluconeogenesis, the TCA cycle, and glutamate metabolism and the urea cycle. Many primary and secondary metabolites generated and released by microorganisms exhibit environment-dependent profiles, and these shifts serve as important indicators of intracellular metabolic changes [23]. *R. sphaeroides* can employ carbon dioxide as a carbon source through its native CBB pathway [20]. The CO_2_ taken up into the cell is subsequently assimilated through glycolysis and the TCA cycle [7]. The identification of glycolytic and TCA cycle intermediates in the extracellular metabolites indicates that the carbon assimilation network connecting the CBB pathway, glycolysis, and the TCA cycle is actively operating within the cells. However, these extracellular metabolite profiles of *R. sphaeroides* observed under autotrophic conditions indicate that the metabolic flux may shift toward overflow metabolism. Overflow metabolism is commonly observed in various microorganisms, such as yeast, bacteria, and mammalian cells, and typically arises under conditions of excess carbon availability or nitrogen limitation [33]. This phenomenon leads to the secretion of metabolic byproducts, including ethanol, acetate, and lactate [33,34]. *R. sphaeroides* has been reported to increase polyhydroxybutyrate (PHB), which known as a carbon storage compound, under carbon-excess or nitrogen-limited conditions [10,35]. An imbalanced C/N ratio reduced target product formation while redirecting carbon flux toward gluconic acid, a major byproduct, for production of bacterial cellulose in *Acetobacter xylinum* [36]. In *C. necator*, alteration of the C/N ratio in culture medium increased the production of 1,3-butanediol [37]. Furthermore, nitrogen limitation also resulted in growth rate, pigment content, and photosynthetic performance, suggesting that it functions as a crucial switch driving metabolic reorganization in microalgae [38]. In addition, redox imbalance can influence overflow metabolism and byproduct formation. Modulation of the intracellular redox state through heterologous expression of NADH oxidase significantly reduced acetate formation in *E. coli*, highlighting the importance of redox balance in controlling overflow metabolism [39]. Overall, our findings suggest that mitigating overflow metabolism in *R. sphaeroides* under autotrophic conditions requires coordinated regulation of the carbon-to-nitrogen ratio as well as intracellular redox balance. These regulations may help minimize the formation of extracellular metabolic byproducts while enhancing biomass accumulation and target product yields.

Based on transcriptomic analysis, we confirmed that autotrophic conditions are associated with a change in central carbon metabolism, accompanied by decreased expression of glycolysis-related genes. In a previous study, transcriptomic profiling in *R. sphaeroides* during the transition from anaerobic photosynthesis to dark-aerobic respiration conditions showed a decreased expression of genes involved in photosynthesis and carbon dioxide fixation, whereas the expression of genes involved in carbohydrate, lipid, and glycerol utilization, as well as aerobic respiration, increased [40,41]. *C. necator* is also a promising CO_2_-fixing microorganism and is recognized as a metabolically versatile species similar to *R. sphaeroides* [42]. Consistent with our results, the expression of glycolytic enzymes and associated transporters was significantly reduced under autotrophic conditions compared to heterotrophic conditions in which fructose was supplied as the carbon source in *C. necator* [43]. Transcriptional upregulation of CBB cycle and C1 metabolism pathways was also observed under autotrophic conditions in *C. necator* [44]. Taken together, these findings indicate that alteration of carbon assimilation strategies under autotrophic conditions are accompanied by transcriptional reorganization of the central carbon metabolism.

Although transcript levels of glycolysis-related genes, including *pgi*, *gapB*, and *lctB*, were reduced under autotrophic conditions, extracellular accumulation of glycolytic intermediates and byproducts, such as lactate, was observed. This discrepancy between transcriptomic and metabolomic profiles may be attributed to various metabolic regulatory mechanisms operating beyond transcriptional control. Previous studies have reported that metabolic regulation is mediated by a multilayered regulatory network involving transcriptional responses, translation, protein degradation, and post-translational modifications [45]. Within this framework, transcriptional regulation primarily governs the allocation of protein resources, whereas post-translational modifications and allosteric protein–metabolite interactions enable the rapid and dynamic fine-tuning of metabolic flux [46]. Furthermore, it is well established that glycolytic flux can be influenced by enzyme–substrate–product interactions and allosteric effectors, as demonstrated in *Saccharomyces cerevisiae* [47]. Taken together, these observations indicate that a single-omics approach is insufficient to explain the complexity of microbial metabolism.

To achieve a more accurate understanding of metabolic interactions and regulatory mechanisms in microorganisms, integrated multi-omics analyses, incorporating genomics, transcriptomics, proteomics, metabolomics, and fluxomics, is required [48]. An integrated multi-omics-based approach enables the identification of metabolic pathway redesign targets, facilitating the development of novel microbial strain engineering strategies [49]. Also, a multi-omics approach can provide systematic insights into cellular physiology and productivity, supporting process understanding and knowledge-driven biomanufacturing design in the production of therapeutic proteins [50]. Recently, standardized multi-omics datasets have been stored in integrated databases to provide foundational training material for machine learning models. This data infrastructure enables acceleration of the Design–Build–Test–Learn (DBTL) cycle in synthetic biology and facilitates the rapid translation of biological insights into biomanufacturing applications [51]. Altogether, an integrative understanding of microbial metabolism under autotrophic conditions may provide a basis for developing strategies to improve the production of target products or biomass in *R. sphaeroides*, while the establishment of a comprehensive multi-omics approach in *R. sphaeroides* may facilitate data-driven strain engineering and enable DBTL-based biomanufacturing development in the future.

## 5. Conclusions

Integrated metabolomics and transcriptomic analyses provide a robust basis for developing carbon dioxide-based biomanufacturing and metabolic engineering strategies. In this study, metabolomic profiling revealed that the detected extracellular metabolites could be classified into three major groups: (i) glycolysis- and gluconeogenesis-related metabolites; (ii) TCA cycle-related metabolites; and (iii) metabolites associated with glutamate metabolism and the urea cycle. Among these, metabolites associated with glycolysis and gluconeogenesis predominated, with lactic acid identified as a major extracellular byproduct. Transcriptomic analysis showed broad the downregulation of glycolytic genes accompanied by the upregulation of the *cfxA* gene. Collectively, these findings indicate a shift in carbon assimilation flux toward CBB cycle-centered metabolism and lactic acid overflow metabolism in *R. sphaeroides* under autotrophic growth conditions.

## Figures and Tables

**Table 1:** Extracellular putative metabolite profiles of *R. sphaeroides* under autotrophic growth conditions.

Related Pathway Description	Mode	Compound Name	*m*/*z* ^1^	MT ^2^	tR ^3^	Relative Area
Glycolysis and Gluconeogenesis	CE-A ^4^	2-deoxy-D-glucose 6-phosphate	243.028	8.34		3.8 × 10^6^
	CE-A	3-Phosphoglyceric acid	184.985	14.10		1.9 × 10^5^
	CE-A	Glucose 6-phosphate	259.020	8.13		7.2 × 10^6^
	CE-A	Glycerol 3-phosphate	171.005	9.76		9.1 × 10^7^
	CE-A	Lactic acid	89.024	8.78		6.5 × 10^5^
	CE-A	Phosphoenolpyruvic acid	166.972	14.78		2.1 × 10^5^
	CE-C ^5^	Glycine	76.040	6.88		7.4 × 10^5^
	CE-C	L-Serine	106.051	8.25		4.6 × 10^5^
	CE-C	L-Threonine	120.064	8.69		1.5 × 10^5^
TCA Cycle for Energy Conversion	CE-A	3-Hydroxybutyric acid	103.039	7.91		1.4 × 10^5^
	CE-A	Mevalonic acid	147.067	7.27		9.6 × 10^6^
	CE-C	L-Alanine	90.056	7.45		4.4 × 10^5^
	CE-C	L-Isoleucine	132.103	8.38		2.2 × 10^5^
	CE-C	L-Leucine	132.103	8.49		2.0 × 10^5^
	CE-C	L-Lysine	147.113	5.67		8.0 × 10^6^
TCA Cycle to Store Energy	CE-C	L-Glutamic acid	148.062	9.04		3.2 × 10^5^
	CE-C	L-Glutamine	147.077	8.89		3.3 × 10^5^
Glutamate Metabolism and Urea Cycle	CE-A	N-Acetylglutamic acid	188.055	10.48		5.4 × 10^6^
	CE-A	5-Oxoproline	128.036	7.84		1.0 × 10^5^
	CE-C	Creatine	132.079	7.30		1.6 × 10^5^
	CE-C	L-Histidine	156.079	6.03		1.1 × 10^5^
	CE-C	L-Proline	116.072	8.89		3.1 × 10^5^
	CE-C	Ornithine	133.098	5.63		2.3 × 10^5^
	CE-C	*S*-Adenosylmethionine	399.148	5.87		1.4 × 10^5^
	CE-C	Spermidine	146.166	3.75		7.1 × 10^6^
	CE-C	Urocanic acid	139.050	6.82		2.8 × 10^5^
Choline Metabolism and Methionine Salvage Pathway	CE-A	Glyoxylic acid	72.993	9.46		1.4 × 10^5^
	CE-C	Trimethylamine	60.081	4.86		1.3 × 10^4^
	CE-C	Trimethylamine *N*-oxide	76.076	5.31		3.6 × 10^5^
Gluconate Shunt Pathway	CE-A	Gluconic acid	195.050	6.86		1.2 × 10^5^
AAA Metabolism—Phenylalanine and Tyrosine	CE-A	3-Phenylpropionic acid	149.060	7.34		8.9 × 10^6^
Fatty Acids pathway	LC-N ^6^	Arachidic acid	311.296		15.93	8.2× 10^7^
	LC-N	Linoleic acid	279.227		14.39	1.5 × 10^6^
	LC-N	Oleic acid	281.247		14.85	5.5 × 10^6^
	LC-N	Palmitic acid	255.233		14.76	4.5 × 10^5^
	LC-N	Stearic acid	283.264		15.38	4.5 × 10^5^
Pyrimidine Metabolism	CE-A	4-Pyridoxic acid	182.046	7.29		7.1 × 10^6^
	CE-A	Thiamine diphosphate	423.031	6.66		1.4 × 10^6^
	CE-C	Nicotinic acid	124.040	8.28		4.7 × 10^4^
	CE-C	Thiamine	265.114	5.42		3.1 × 10^5^
Others	CE-A	2-Hydroxy-4-methylvaleric acid	131.073	7.34		3.1 × 10^6^
	CE-A	3-Hydroxyphenylacetic acid	151.040	7.25		1.3 × 10^4^
	CE-A	Indole-3-carboxylic acid	160.039	7.36		8.6 × 10^6^
	CE-A	Methyl sulfate	110.975	11.96		1.1 × 10^5^
	CE-A	N^2^-Acetylaminoadipic acid	202.071	9.89		6.1 × 10^6^
	CE-A	N-Acetylmuramic acid	292.104	6.16		6.9 × 10^6^
	CE-A	N-Ethylmaleimide+H_2_O	142.050	7.44		3.5 × 10^6^
	CE-A	*p*-Toluic acid	135.045	7.63		3.5 × 10^5^
	CE-A	Vanillic acid	167.035	7.35		3.9 × 10^6^
	CE-C	Aminoacetone	74.061	5.30		1.4 × 10^4^
	CE-C	Cadaverine	103.123	4.12		2.8 × 10^5^
	CE-C	Dimethylaminoethanol	90.092	5.73		2.0 × 10^5^
	CE-C	Glycerol	93.055	18.37		1.2 × 10^3^
	CE-C	Isopropanolamine	76.076	5.77		8.7 × 10^6^
	CE-C	Phenol	95.047	4.39		2.0 × 10^4^
	CE-C	Pterin	164.056	8.60		1.2 × 10^5^
	LC-N	Myristic acid	227.202		14.04	6.6 × 10^6^
	LC-P ^7^	1,2-Dipalmitoyl-(D/L)-glycero-3-phosphoethanolamine	692.520		16.69	1.2 × 10^5^
	LC-P	Corosolic acid	455.345		13.15	1.2 × 10^4^
	LC-P	Indole-3-carboxaldehyde	146.060		7.00	6.6 × 10^5^
	LC-P	Oleoyl ethanolamide	326.304		14.32	1.7 × 10^5^
	LC-P	Retinol	269.229		13.76	2.1 × 10^5^
	LC-P	Sitosterol	397.385		16.57	9.4 × 10^6^
	LC-P	Stearoyl ethanolamide	328.321		14.60	1.9 × 10^5^
	LC-P	α-Tocopherol acetate	490.424		16.67	9.7 × 10^6^

^1^ *m*/*z*: This denotes the mass-to-charge ratio, a fundamental measurement in mass spectrometry. ^2^ MT: This stands for ‘migration time’ and is a key parameter in capillary electrophoresis time-of-flight mass spectrometry. ^3^ tR: This stands for ‘retention time’ and is a key parameter in liquid chromatography time-of-flight mass spectrometry. ^4^ CE-A: CE-TOFMS(anion). This indicates that capillary electrophoresis–time-of-flight mass spectrometry was operated in negative ion mode. This mode is specifically used to analyze hydrophilic and highly polar metabolites that carry a negative charge. ^5^ CE-C: CE-TOFMS(cation). This indicates that capillary electrophoresis–time-of-flight mass spectrometry was operated in positive ion mode. This mode is specifically used to analyze hydrophilic and highly polar metabolites that carry a positive charge. ^6^ LC-N: LC-TOFMS(negative). This indicates that liquid chromatography–time-of-flight mass spectrometry was operated in negative mode. This mode is primarily used for detecting lipophilic and relatively non-polar metabolites that ionize favorably by acquiring a negative charge. ^7^ LC-P: LC-TOFMS(positive). This indicates that liquid chromatography–time-of-flight mass spectrometry was operated in positive mode. This mode is primarily used for detecting lipophilic and relatively non-polar metabolites that ionize favorably by acquiring a positive charge.

**Table 2:** Quantitative estimation of extracellular metabolites produced by *R. sphaeroides*.

Related Pathway Description	Mode	Compound Name	Concentration (µM)
Glycolysis and Gluconeogenesis	CE-A ^1^	3-Phosphoglyceric acid	1.70
	CE-A	Glucose 6-phosphate	0.50
	CE-A	Glycerol 3-phosphate	0.08
	CE-A	Glycine	2.20
	CE-A	Threonine	0.30
	CE-C ^2^	Lactic acid	4.90
	CE-C	Serine	1.20
	LC-N ^3^	Phosphoenolpyruvic acid	1.60
TCA Cycle for Energy Conversion	CE-A	3-Hydroxybutyric acid	0.90
	CE-C	Alanine	0.80
	CE-C	Isoleucine	0.20
	CE-C	Leucine	0.14
	CE-C	Lysine	0.15
TCA Cycle to Store Energy	CE-C	Glutamic acid	0.80
	CE-C	Glutamine	0.80
Glutamate Metabolism and Urea Cycle	CE-C	Creatine	0.20
	CE-C	Histidine	0.20
	CE-C	Ornithine	0.40
	CE-C	Proline	0.40
	CE-C	*S*-Adenosylmethionine	0.40
	CE-C	Spermidine	0.08
Choline Metabolism and Methionine Salvage Pathway	CE-A	Glyoxylic acid	2.80
Gluconate shunt pathway	CE-A	Gluconic acid	0.70

^1^ CE-A: CE-TOFMS(anion). This indicates that capillary electrophoresis time-of-flight mass spectrometry was operated in negative ion mode. This mode is specifically used to analyze hydrophilic and highly polar metabolites that carry a negative charge. ^2^ CE-C: CE-TOFMS(cation). This indicates that capillary electrophoresis time-of-flight mass spectrometry was operated in positive ion mode. This mode is specifically used to analyze hydrophilic and highly polar metabolites that carry a positive charge. ^3^ LC-N: LC-TOFMS(negative). This indicates that liquid chromatography time-of-flight mass spectrometry was operated in negative mode. This mode is primarily used for detecting lipophilic and relatively non-polar metabolites that ionize favorably by acquiring a negative charge.

**Table 3:** Comparison of transcript levels between autotrophic and heterotrophic conditions.

Gene Number	Gene	Function		Log_2_(FC)
RSP_2736	*pgi*	Glucose-6-phosphate isomerase		−1.5
RSP_1283	*cfxA*	Fructose-1,6-bisphosphate aldolase		5.0
RSP_4045	*fbaB*	Fructose-bisphosphate aldolase		−1.4
RSP_2959	*gapB*	Glyceraldehyde-3-phosphate dehydrogenase		−2.4
RSP_4044	*pgk*	Phosphoglycerate kinase		−1.2
RSP_0934	*gpmI*	2,3-bisphosphoglycerate-independent phosphoglycerate mutase		−1.2
RSP_2491	*eno*	Enolase		−2.1
RSP_1766	*pykA*	Pyruvate kinase		−1.6
RSP_0829	*lctB*	Lactate dehydrogenase		−1.5

## Data Availability

The original contributions presented in this study are included in the article. Further inquiries can be directed to the corresponding authors.

## Supplementary Materials

The following supporting information can be downloaded at: https://www.mdpi.com/article/10.3390/metabo16030156/s1, Table S1: Transcriptional comparison of *R. sphaeroides* under autotrophic versus heterotrophic conditions.; Figure S1. Gene ontology classification of differentially expressed genes.

## Abbreviations

The following abbreviations are used in this manuscript:

GtCO_2_	Gigatons of Carbon Dioxide
CBB cycle	Calvin–Benson–Bassham cycle
MEP	Methylerythritol 4-phosphate
CE-TOFMS	Capillary Electrophoresis–Time-of-Flight Mass Spectrometry
LC-TOFMS	Liquid Chromatography–Time-of-Flight Mass Spectrometry
CE-C	Cation (mode in CE-TOFMS)
CE-A	Anion (mode in CE-TOFMS)
LC-N	Negative (mode in LC-TOFMS)
LC-P	Positive (mode in LC-TOFMS)
ODS	Octadecylsilyl (column)
*m*/*z*	Mass-to-Charge Ratio
MT	Migration Time
tR	Retention Time
C/N	Carbon-to-Nitrogen (ratio)
DBTL	Design–Build–Test–Learn
OD660	Optical Density at 660 nm
FPKM	Fragments Per Kilobase of transcript per Million mapped reads
RPKM	Reads Per Kilobase of transcript per Million mapped reads
GO	Gene Ontology
TCA	Tricarboxylic Acid (cycle)
LoD	Limit of Detection
S/N	Signal-to-Noise Ratio Criteria

## References

[1] Liu Z., Deng Z., Davis S.J., Ciais P. (2024). Global carbon emissions in 2023. Nat. Rev. Earth Environ..

[2] Onyeaka H., Ekwebelem O.C. (2023). A Review of Recent Advances in Engineering Bacteria for Enhanced CO_2_ Capture and Utilization. Int. J. Environ. Sci. Technol..

[3] Michele A., Dibenedetto A. (2020). Carbon Recycling through CO_2_-Conversion for Stepping toward a Cyclic-C Economy. A Perspective. Front. Energy Res..

[4] Pikaar I., de Vrieze J., Rabaey K., Herrero M., Smith P., Verstraete W. (2018). Carbon Emission Avoidance and Capture by Producing in-Reactor Microbial Biomass Based Food, Feed and Slow Release Fertilizer: Potentials and Limitations. Sci. Total Environ..

[5] Decker S.R., Brunecky R., Yarbrough J.M., Subramanian V. (2023). Perspectives on biorefineries in microbial production of fuels and chemicals. Front. Ind. Microbiol..

[6] Bachleitner S., Ata O., Mattanovich D. (2023). The potential of CO_2_-based production cycles in biotechnology to fight the climate crisis. Nat. Commun..

[7] Orsi E., Beekwilder J., Eggink G., Kengen S.W.M., Weusthuis R.A. (2021). The transition of *Rhodobacter sphaeroides* into a microbial cell factory. Biotechnol. Bioeng..

[8] Wang Q., Quan S., Xiao H. (2019). Towards efficient terpenoid biosynthesis: Manipulating IPP and DMAPP supply. Bioresour. Bioprocess..

[9] Mougiakos I., Orsi E., Ghiffary M.R., Post W., de Maria A., Adiego-Perez B., Kengen S.W.M., Weusthuis R.A., van der Oost J. (2019). Efficient Cas9-based genome editing of *Rhodobacter sphaeroides* for metabolic engineering. Microb. Cell Fact..

[10] Orsi E., Mougiakos I., Post W., Beekwilder J., Dompe M., Eggink G., van der Oost J., Kengen S.W.M., Weusthuis R.A. (2020). Growth-uncoupled isoprenoid synthesis in *Rhodobacter sphaeroides*. Biotechnol. Biofuels.

[11] Carruthers D.N., Lee T.S. (2021). Diversifying Isoprenoid Platforms via Atypical Carbon Substrates and Non-model Microorganisms. Front. Microbiol..

[12] Eiben C.B., de Rond T., Bloszies C., Gin J., Chiniquy J., Baidoo E.E.K., Petzold C.J., Hillson N.J., Fiehn O., Keasling J.D. (2019). Mevalonate Pathway Promiscuity Enables Noncanonical Terpene Production. ACS Synth. Biol..

[13] Lee S., Rim Lee Y., Lee W.H., Youn Lee S., Moon M., Woo Park G., Min K., Lee J., Lee J.S. (2022). Valorization of CO_2_ to beta-farnesene in *Rhodobacter sphaeroides*. Bioresour. Technol..

[14] Zhang H., HuangFu H., Qin G., Wu G., Wang L., Tan Z. (2023). Transcriptomic and metabolomic insights into the antimicrobial effect of *Leuconostoc mesenteroides* or lactic acid on pathogenic *Gallibacterium anatis*. Chem. Biol. Technol. Agric..

[15] Lim J., Park C., Kim M., Kim H., Kim J., Lee D.S. (2024). Advances in single-cell omics and multiomics for high-resolution molecular profiling. Exp. Mol. Med..

[16] Amer B., Baidoo E.E.K. (2021). Omics-Driven Biotechnology for Industrial Applications. Front. Bioeng. Biotechnol..

[17] Salusjarvi L., Ojala L., Peddinti G., Lienemann M., Jouhten P., Pitkanen J.P., Toivari M. (2022). Production of biopolymer precursors beta-alanine and L-lactic acid from CO_2_ with metabolically versatile *Rhodococcus opacus* DSM 43205. Front. Bioeng. Biotechnol..

[18] Sistrom W.R. (1960). A Requirement for Sodium in the Growth of *Rhodopseudomonas spheroides*. Microbiology.

[19] Lee Y.R., Lee W.H., Lee S.Y., Lee J., Kim M.S., Moon M., Park G.W., Kim H.S., Kim J.I., Lee J.S. (2022). Regulation of Reactive Oxygen Species Promotes Growth and Carotenoid Production Under Autotrophic Conditions in *Rhodobacter sphaeroides*. Front. Microbiol..

[20] Lee Y.R., Lee S.Y., Lee J., Kim H.S., Lee J.-S., Lee W.-H., Lee S. (2022). Modulation of Antioxidant Activity Enhances Photoautotrophic Cell Growth of *Rhodobacter sphaeroides* in Microbial Electrosynthesis. Energies.

[21] Shin J., Yang J., Cha E., Kim H., Lee Y., Kim S., Choi I., Yang J. (2021). Analyzing the Metabolomic Profile of Yellowtail (*Seriola quinquerdiata*) by Capillary Electrophoresis-Time of Flight Mass Spectrometry to Determine Geographical Origin. Metabolites.

[22] Suzuki M., Yoshioka M., Ohno Y., Akune Y. (2018). Plasma metabolomic analysis in mature female common bottlenose dolphins: Profiling the characteristics of metabolites after overnight fasting by comparison with data in beagle dogs. Sci. Rep..

[23] Pinu F.R., Villas-Boas S.G. (2017). Extracellular Microbial Metabolomics: The State of the Art. Metabolites.

[24] Verpoorte R., Kim H.K., Choi Y.H. (2022). Trivialities in metabolomics: Artifacts in extraction and analysis. Front. Mol. Biosci..

[25] Langner M., Frobel D., Helm J., Chavakis T., Peitzsch M., Bechmann N. (2024). Accurate redox state indication by in situ derivatization with N-ethylmaleimide—Profiling of transsulfuration and glutathione pathway metabolites by UPLC-MS/MS. J. Chromatogr. B Anal. Technol. Biomed. Life Sci..

[26] Carthew R.W. (2021). Gene Regulation and Cellular Metabolism: An Essential Partnership. Trends Genet..

[27] Lucius S., Hagemann M. (2024). The primary carbon metabolism in cyanobacteria and its regulation. Front. Plant Sci..

[28] Paoletti M.M., Fournier G.P. (2022). Chimeric inheritance and crown-group acquisitions of carbon fixation genes within Chlorobiales: Origins of autotrophy in Chlorobiales and implication for geological biomarkers. PLoS ONE.

[29] Tang M., Zhen X., Zhao G., Wu S., Hua W., Qiang J., Yanling C., Wang W. (2024). The metabolic pathways of carbon assimilation and polyhydroxyalkanoate production by *Rhodospirillum rubrum* in response to different atmospheric fermentation. PLoS ONE.

[30] Godoy M.S., de Miguel S.R., Prieto M.A. (2023). Aerobic-anaerobic transition boosts poly(3-hydroxybutyrate-co-3-hydroxyvalerate) synthesis in *Rhodospirillum rubrum*: The key role of carbon dioxide. Microb. Cell Factories.

[31] Kim S., Jang Y.J., Gong G., Lee S.M., Um Y., Kim K.H., Ko J.K. (2022). Engineering *Cupriavidus necator* H16 for enhanced lithoautotrophic poly(3-hydroxybutyrate) production from CO_2_. Microb. Cell Factories.

[32] Li Z., Xin X., Xiong B., Zhao D., Zhang X., Bi C. (2020). Engineering the Calvin-Benson-Bassham cycle and hydrogen utilization pathway of *Ralstonia eutropha* for improved autotrophic growth and polyhydroxybutyrate production. Microb. Cell Factories.

[33] Paczia N., Nilgen A., Lehmann T., Gätgens J., Wiechert W., Noack S. (2012). Extensive exometabolome analysis reveals extended overflow metabolism in various microorganisms. Microb. Cell Factories.

[34] Gosselin-Monplaisir T., Enjalbert B., Uttenweiler-Joseph S., Portais J.C., Heux S., Millard P. (2025). Overflow metabolism in bacterial, yeast, and mammalian cells: Different names, same game. Mol. Syst. Biol..

[35] Lee Y.R., Fitriana H.N., Lee S.Y., Kim M.-S., Moon M., Lee W.-H., Lee J.-S., Lee S. (2020). Molecular Profiling and Optimization Studies for Growth and PHB Production Conditions in *Rhodobacter sphaeroides*. Energies.

[36] Lahiri D., Nag M., Dutta B., Dey A., Sarkar T., Pati S., Edinur H.A., Abdul Kari Z., Mohd Noor N.H., Ray R.R. (2021). Bacterial Cellulose: Production, Characterization, and Application as Antimicrobial Agent. Int. J. Mol. Sci..

[37] Weiler J.R., Jurgensen N., Cornejo Infante M., Knoll M.T., Gescher J. (2024). Strain and model development for auto- and heterotrophic 2,3-butanediol production using *Cupriavidus necator* H16. Biotechnol. Biofuels Bioprod..

[38] Li X., Li W., Zhai J., Wei H. (2018). Effect of nitrogen limitation on biochemical composition and photosynthetic performance for fed-batch mixotrophic cultivation of microalga *Spirulina platensis*. Bioresour. Technol..

[39] Vemuri G.N., Altman E., Sangurdekar D.P., Khodursky A.B., Eiteman M.A. (2006). Overflow metabolism in *Escherichia coli* during steady-state growth: Transcriptional regulation and effect of the redox ratio. Appl. Environ. Microbiol..

[40] Arai H., Roh J.H., Kaplan S. (2008). Transcriptome dynamics during the transition from anaerobic photosynthesis to aerobic respiration in *Rhodobacter sphaeroides* 2.4.1. J. Bacteriol..

[41] Bathke J., Konzer A., Remes B., McIntosh M., Klug G. (2019). Comparative analyses of the variation of the transcriptome and proteome of *Rhodobacter sphaeroides* throughout growth. BMC Genom..

[42] Wang Y., Cui L., Ding L., Su X., Luo H., Huang H., Wang Y., Yao B., Zhang J., Wang X. (2024). Unlocking the potential of *Cupriavidus necator* H16 as a platform for bioproducts production from carbon dioxide. World J. Microbiol. Biotechnol..

[43] Pu X., Weng C., Li Y., Geng B., Yang H., Peng X., Han Y. (2026). Engineering Mixotrophy in the Chemolithoautotrophic *Cupriavidus necator* through Hydrogenase Induction. ACS Synth. Biol..

[44] Salinas A., McGregor C., Irorere V., Arenas-Lopez C., Bommareddy R.R., Winzer K., Minton N.P., Kovacs K. (2022). Metabolic engineering of *Cupriavidus necator* H16 for heterotrophic and autotrophic production of 3-hydroxypropionic acid. Metab. Eng..

[45] Chubukov V., Gerosa L., Kochanowski K., Sauer U. (2014). Coordination of microbial metabolism. Nat. Rev. Microbiol..

[46] Kochanowski K., Sauer U., Noor E. (2015). Posttranslational regulation of microbial metabolism. Curr. Opin. Microbiol..

[47] Daran-Lapujade P., Rossell S., van Gulik W.M., Luttik M.A.H., de Groot M.J.L., Slijper M., Heck A.J.R., Daran J.-M., de Winde J.H., Westerhoff H.V. (2007). The fluxes through glycolytic enzymes in *Saccharomyces cerevisiae* are predominantly regulated at posttranscriptional levels. Proc. Natl. Acad. Sci. USA.

[48] Reaves M.L., Rabinowitz J.D. (2011). Metabolomics in systems microbiology. Curr. Opin. Biotechnol..

[49] John P.C., Bomble Y.J. (2019). Approaches to Computational Strain Design in the Multiomics Era. Front. Microbiol..

[50] Wan S., Liu X., Sun W., Lv B., Li C. (2023). Current advances for omics-guided process optimization of microbial manufacturing. Bioresour. Bioprocess..

[51] Ramzi A.B., Baharum S.N., Bunawan H., Scrutton N.S. (2020). Streamlining Natural Products Biomanufacturing with Omics and Machine Learning Driven Microbial Engineering. Front. Bioeng. Biotechnol..

