# Supplementary material for: Extracellular Metabolite Profiling in CO2-Fixing Bacterium Rhodobacter sphaeroides Under Autotrophic Conditions
Source: Metabolites. 2026 Feb 26;16(3):156. doi: 10.3390/metabo16030156 (PMC13027526; doi:10.3390/metabo16030156)
Supplement: Supplementary file 1 [file metabolites-16-00156-s001.zip › metabolites-4144321-supplementary.pdf]

Table S1. Transcriptional comparison of *R. sphaeroides* under autotrophic versus heterotrophic conditions. This table presents a selection of differentially expressed genes identified from RNA sequencing analysis in *R. sphaeroides* when grown under autotrophic conditions compared to heterotrophic conditions.

RSP_ID	Gene	Function	log_2(FC)
RSP_0496	*hoxL*	Hydrogenase protein large subunit	11.2
RSP_0495	*hoxK*	Hydrogenase protein small subunit	10.1
RSP_0497	.	HupE/UreJ accessory protein	9.1
RSP_0499	*hyaD*	Hydrogenase 1 maturation peptidase HyaD	8.6
RSP_0503	*hrb*	HupJ, contains rubredoxin domain	6.8
RSP_0502	.	HupH hydrogenase expression/formation protein	6.7
RSP_1784	*pdtaR*	Response regulator receiver domain protein	6.3
RSP_1285	*fbp*	Fructose-1,6-bisphosphatase class 1 1	6.0
RSP_1284	*prkA*	Phosphoribulokinase	5.9
RSP_0505	*hybF*	Hydrogenase maturation factor HypA	5.9
RSP_6025	.	Uncharacterized protein	5.7
RSP_0960	*ccrA2*	Crotonyl-CoA carboxylase/reductase	5.2
RSP_0508	*hybG*	Hydrogenase maturation protein HypC	5.2
RSP_0507	*hupR1*	HupR response regulator	5.2
RSP_0506	*hypB*	Hydrogenase nickel incorporation protein HypB	5.1
RSP_2800	*acoR*	AcoR, Transcriptional activator of acetoin/glycerol metabolism	5.0
RSP_0509	*hypD*	Hydrogenase maturation factor	5.0
RSP_1283	*cfxA*	Fructose-1,6-bisphosphate aldolase	5.0
RSP_2232	*udg_1*	Type-4 uracil-DNA glycosylase	4.8
RSP_0510	*hypE*	Hydrogenase maturation protein, carbamoyl dehydratase HypE	4.7
RSP_0415	*sigE*	RNA polymerase, sigma subunit, ECF family	4.4
RSP_1933	.	Outer membrane protein, OmpA/MotB family	4.4
RSP_1270	.	CsbD domain-containing protein	4.4
DQL45_10305	*dnaE2_1*	Uncharacterized protein	4.4
RSP_2375	.	Protein adenylyltransferase SelO	4.4
RSP_2115	*lpxC*	UDP-3-O-acyl-N-acetylglucosamine deacetylase	4.3
RSP_2641	.	Uncharacterized protein	4.3
RSP_0753	.	Salt-stress induced outer membrane protein	4.2
RSP_2575	*gfa*	Glutathione-dependent formaldehyde-activating enzyme	4.1
RSP_1016	*ibpA_2*	Small heat shock protein	4.1
RSP_0399	.	Tad domain-containing protein	4.0
RSP_0523	*ku_2*	Non-homologous end joining protein Ku	4.0
RSP_2378	*cypC*	Putative fatty acid beta hydroxylase (Cytochrome P450)	4.0
RSP_0400	.	TadE-like protein	4.0
RSP_0988	*frcA_2*	Fructose ABC transporter ATP-binding proteinribose	4.0
RSP_2305	*phaJ_1*	MaoC family protein	3.9
RSP_1473	.	Uncharacterized protein	3.9
RSP_0961	*mutB*	Ethylmalonyl-CoA mutase	3.9

RSP_0989	*frcC*	Mannose ABC transporter membrane proteinribose	3.9
RSP_0990	*frcB*	Ribose-binding proteinmannose-binding proteinfructose-binding protein	3.8
RSP_0766	*ppk2*	Polyphosphate kinase	3.7
RSP_0947	.	Protein of unknwon function (DUF3008)	3.7
RSP_2121	.	DUF1638 domain-containing protein	3.6
RSP_1458	*imuB*	DNA repair enzyme	3.6
RSP_2888	*nsrR*	Transcriptional regulator, BadM/Rrf2 family	3.6
RSP_2135	.	RHH_4 domain-containing protein	3.5
RSP_2234	.	Putative DNA-binding protein	3.5
RSP_0949	*otsB*	Trehalose 6-phosphate phosphatase	3.5
RSP_2578	*moxF*	Putative pqq dehydrogenase protein	3.5
RSP_2579	*cycB*	Cytochrome c553i	3.5
RSP_1609	*uxaB*	Altronate dehydrogenase	3.4
RSP_1551	.	Putative glyoxalase family protein	3.4
RSP_1282	*cbbL*	Ribulose bisphosphate carboxylase large chain	3.4
RSP_2274	*livF_3*	Amino acid/amide ABC transporter ATP-binding protein 2, HAAT family	3.4
RSP_2976	.	Putative integral membrane protein	3.4
RSP_0424	.	Uncharacterized protein	3.3
RSP_2338	.	PRC-barrel domain-containing protein	3.3
RSP_0521	*adh_1*	Putative Zn-containing dehydrogenase	3.3
RSP_2961	*hrp1_2*	Protein containing a CBS domain	3.3
RSP_1281	*cbbS*	Ribulose 1,5-bisphosphate carboxylase small subunit	3.3
RSP_0578	*adk_1*	Adenylate kinase	3.3
RSP_6002	.	Uncharacterized protein	3.2
RSP_0520	*wbiB*	NAD-dependent dehydratase/epimerase	3.2
RSP_2640	.	Putative membrane protein/domain protein	3.2
RSP_2276	.	Amino acid/amide ABC transporter membrane protein 2, HAAT family	3.2
RSP_0601	*rpoH_1*	RNA polymerase, sigma 32 subunit, RpoH	3.2
RSP_2679	*ligD*	3'-phosphoesterase	3.2
RSP_2677	*mgtC*	Protein MgtC (putative Mg2+ transporter-C (MgtC) family protein)	3.2
RSP_2278	*cysA_4*	Amino acid/amide ABC transporter ATP-binding protein 1, HAAT family	3.1
RSP_1331	.	Flagellar basal body protein	3.1
RSP_2384	.	Putative stress protein (General stress protein 26)	3.1
RSP_2660	.	Ribonuclease BN family protein	3.1
RSP_2277	*livH_3*	Amino acid/amide ABC transporter membrane protein 1, HAAT family	3.1
RSP_3609	*spo0C_2*	ParB-like nuclease	3.1
RSP_1608	*lgoD_2*	Zn-dependent dehydrogenase	3.1
RSP_0401	.	Flp pilus assembly protein TadG	3.0

RSP_2890	*actP*	Copper-translocating P-type ATPase (P-type Cu+ transporter)	3.0
RSP_2740	.	Putative Glucose/sorbosone dehydrogenase	3.0
RSP_1992	.	DUF2235 domain-containing protein	3.0
RSP_0028	*sadH*	Short chain dehydrogenase	3.0
RSP_1330	*flgC_2*	Putative flagellar basal-body rod protein FlgC	3.0
RSP_2681	*ecfG_1*	RNA polymerase sigma factor	3.0
RSP_2871	*araQ_1*	Maltose ABC transporter membrane proteintrehalose	3.0
RSP_1610	*garD*	Altronate hydrolase	3.0
RSP_2392	.	Putative membrane protein, similar to periplasmic nitrate reductase NnuR	2.9
RSP_0870	.	Uncharacterized protein	2.9
RSP_0414	.	Putative integral membrane protein	2.9
RSP_1171	.	DNA repair protein	2.9
RSP_2676	.	Putative periplasmic or secreted lipoprotein	2.9
RSP_1695	.	SH3 domain-containing protein	2.9
RSP_1280	*cbbX*	CbbX protein	2.9
Rsph17029_2105	*recA*	Protein RecA	2.9
RSP_1907	.	Flp pilus assembly protein ATPase CpaE	2.9
RSP_2275	.	Amino acid/amide ABC transporter substrate-binding protein, HAAT family	2.9
RSP_0531	*iscU*	Nitrogen fixation protein NifU	2.9
RSP_2273	.	Putative anaerobic phenylacetate CoA ligase	2.9
RSP_0554	*htpX*	Protease HtpX homolog (Heat shock protein)	2.8
RSP_2779	*katA*	Catalase	2.8
RSP_1139	.	Transcriptional regulator, MarR family	2.8
RSP_1083	*arcA_2*	Two component transcriptional regulator, winged helix family	2.8
RSP_0869	.	Response regulator receive protein	2.8
RSP_2872	*lacF*	Maltose ABC transporter membrane proteintrehalose	2.8
RSP_2870	*malK_2*	Maltose ABC transporter ATP-binding proteintrehalose	2.8
RSP_6097	.	Hint_2 domain-containing protein	2.8
RSP_2874	*bglA*	Beta-glucosidase	2.8
RSP_2279	.	Long chain acyl-CoA synthetase	2.7
RSP_0524	*ku_1*	Non-homologous end joining protein Ku	2.7
RSP_1565	.	AppA, antirepressor of ppsR, sensor of blue light	2.7
RSP_1500	.	Putative anti-sigma regulatory kinase	2.7
RSP_0133	*mleN*	Transporter, NhaC family	2.7
RSP_2472	.	Uncharacterized protein	2.7
RSP_2471	.	Putative phage phi-C31 gp36 major capsid-like protein	2.7
RSP_0413	*pleD*	Response regulator receiver modulated diguanylate cyclase	2.6
RSP_2140	.	Uncharacterized protein	2.6
RSP_2387	.	Uncharacterized protein	2.6
RSP_1552	*allA*	Ureidoglycolate hydrolase	2.6

RSP_0118	*ctaC_1*	Cytochrome c oxidase, subunit IIc	2.6
RSP_0813	*phoP_1*	Response regulator receiver protein	2.6
RSP_2590	.	Putative secreted protein	2.6
RSP_1549	*yebE_1*	Uncharacterized protein	2.6
RSP_2998	.	Phage terminase-like protein, large subunit	2.6
RSP_1304	.	Flagellar hook-associated protein 1	2.5
RSP_2123	.	Radical SAM domain protein	2.5
RSP_2380	*katE*	Catalase	2.5
RSP_0530	*nifS*	Cysteine desulfurase	2.5
RSP_2310	*groS1*	10 kDa chaperonin	2.5
RSP_1329	*fliE_2*	Putative flagellar hook-basal body complex protein	2.5
RSP_1820	*yciF_2*	DUF892 domain-containing protein	2.5
RSP_2719	*lrpC*	Transcriptional regulator, AsnC/Lrp family	2.5
RSP_2673	*gcd*	Quinoprotein glucose dehydrogenase	2.5
RSP_2425	*carD*	Transcriptional regulator, CarD family	2.5
RSP_0946	.	Putative DNA topoisomerase I protein	2.5
RSP_7578	*hvrA_3*	H-NS histone family protein	2.5
RSP_0461	*mdtA_1*	Multidrug/cation efflux pump, membrane fusion protein (MFP) family	2.5
RSP_1607	*lgoR*	Transcriptional regulator, GntR family	2.5
RSP_6081	.	Uncharacterized protein	2.5
RSP_1521	*divL*	Uncharacterized protein	2.5
RSP_2570	*yraA*	Protease I	2.5
RSP_0066	*flhB_1*	Flagellar biosynthetic protein FlhB	2.4
RSP_0529	*leuA_1*	Homocitrate synthase	2.4
RSP_0820	.	Cytochrome B561	2.4
RSP_6114	.	Heme-dependent protein with NO-binding domain	2.4
RSP_0864	.	SOUL heme-binding protein	2.4
RSP_2749	*intA*	Putative P4-family integrase	2.4
RSP_2559	*hepA*	ABC transporter, fused ATPase and inner membrane subunits	2.4
RSP_1796	*sodC*	Superoxide dismutase [Cu-Zn]	2.4
RSP_1771	*mcl1*	L-malyl-CoA/beta-methylmalyl-CoA lyase	2.4
RSP_2078	.	Uncharacterized protein	2.4
RSP_1274	*phyR_2*	Two-component response regulator	2.4
RSP_1279	*cbbY*	CbbY family protein	2.4
RSP_0572	*polC*	DNA-directed DNA polymerase	2.4
RSP_2421	.	TPR_REGION domain-containing protein	2.3
RSP_3764	.	Uncharacterized protein	2.3
RSP_0235	*moaA*	GTP 3',8-cyclase	2.3
RSP_0585	*osmC*	Osmotically inducible protein OsmC (peroxidase)	2.3
RSP_1278	*cbbZC*	Phosphoglycolate phosphatase	2.3
RSP_1840	.	Uncharacterized protein	2.3

RSP_2741	.	Class I diheme cytochrome c4	2.3
RSP_1078	*hndA*	Formate dehydrogenase gamma subunit	2.3
RSP_2663	.	Carbohydrate ABC transporter substrate-binding protein, CUT1 family	2.3
RSP_2363	*gno_1*	Short-chain dehydrogenase/reductase	2.3
RSP_1573	.	Putative small protein	2.3
RSP_0068	*rpoN1*	RNA polymerase sigma-54 factor	2.3
RSP_1313	.	Flagellar protein FliL	2.2
RSP_2445	*glgE1*	Alpha-1,4-glucan:maltose-1-phosphate maltosyltransferase	2.2
RSP_2186	*pccR*	Propionyl-CoA carboxylase regulator	2.2
RSP_0088	*yciF_1*	YcfI, putative structural proteins	2.2
RSP_2335	.	Trp repressor binding protein WrbA, putative	2.2
RSP_1867	*decR_2*	Transcriptional regulator, AsnC family	2.2
RSP_0541	*nifH*	Nitrogenase iron protein	2.2
RSP_1572	*ibpA_1*	Heat shock protein, Hsp20 family	2.2
RSP_0611	*hxlR*	Transcriptional regulator, HxlR family	2.2
RSP_2227	*glgC_2*	ADP-glucose pyrophosphorylase	2.2
RSP_1348	.	Putative membrane protein	2.1
RSP_0936	*dapF*	Diaminopimelate epimerase	2.1
RSP_0991	.	Transcriptional regulator, ROK family	2.1
RSP_0860	.	Endo/exonuclease/phosphatase domain-containing protein	2.1
RSP_2556	.	Transglut_core3 domain-containing protein	2.1
RSP_0927	*dmlR_3*	Transcriptional regulator, LysR family	2.1
RSP_1873	*ycfS*	YkuD domain-containing protein	2.1
RSP_6134	.	Uncharacterized protein	2.1
RSP_6079	.	Uncharacterized protein	2.1
RSP_2125	.	Uncharacterized protein	2.1
RSP_2580	.	Amino acid ABC transporter substrate-binding protein, PAAT family	2.1
RSP_1312	*fliF_2*	Flagellar M-ring protein	2.1
RSP_0573	*phoP_2*	Response regulator receiver protein	2.1
RSP_1258	*xylF_1*	Putative hydrolase	2.1
RSP_0591	*cspA_1*	Cold-shock DNA-binding protein family	2.1
RSP_2311	*groL1*	60 kDa chaperonin 1	2.1
RSP_2793	*prmB*	Reductase component of monooxygenase	2.1
RSP_1905	.	Flp pilus assembly protein ATPase CpaF	2.1
RSP_6218	.	Putative conserved small protein containing a coiled-coil domain	2.0
RSP_0686	*clpS*	ATP-dependent Clp protease adapter protein ClpS	2.0
RSP_2730	*sdpR_1*	Transcriptional regulator, ArsR family	2.0
RSP_0937	*treS_2*	Alpha amylase, catalytic subdomain protein	2.0
RSP_1259	.	PLDc_N domain-containing protein	2.0
RSP_1314	.	Uncharacterized protein	2.0

RSP_1563	*limB*	Putative oxidoreductase	2.0
RSP_1315	.	Uncharacterized protein	2.0
RSP_0475	.	Cytochrome b	2.0
RSP_2434	*cheR_1*	Chemotaxis protein methyltransferase	2.0
RSP_1436	*yheS_2*	ABC transporter, duplicated ATPase domains	2.0
RSP_1786	*ptsI*	Phosphocarrier protein HPr	2.0
RSP_1435	*acuR*	Transcriptional regulator AcuR	2.0
RSP_0547	*nifA*	Nif-specific regulatory protein	2.0
RSP_1213	*yebE_2*	Uncharacterized protein	2.0
RSP_1378	.	Uncharacterized protein	2.0
RSP_2879	.	Uncharacterized protein	2.0
RSP_1759	.	Uncharacterized protein	2.0
RSP_0575	*hrp1_1*	Putative signal transduction protein containing cyclic nucleotide-binding and CBS domains	2.0
RSP_1791	*zntA*	Heavy metal-(Cd/Co/Hg/Pb/Zn)-translocating P-type ATPase	2.0
RSP_1486	*yxaF*	Transcriptional regulator, TetR family	2.0
RSP_0948	*otsA*	Trehalose-6-phosphate synthase	1.9
RSP_1472	.	Uncharacterized protein	1.9
RSP_0217	*intS*	Putative phage-related integrase	1.9
RSP_1272	*ecfG_2*	RNA polymerase sigma factor	1.9
RSP_0537	*nifK_2*	Nitrogenase iron-molybdenum cofactor biosynthesis protein NifN	1.9
RSP_1138	*ompR_2*	Two component transcriptional regulator, winged helix family	1.9
RSP_1104	.	Uncharacterized protein	1.9
RSP_0538	*nifD_2*	Nitrogenase iron-molybdenum cofactor biosynthesis protein NifE	1.9
RSP_1164	.	Putative nucleic-acid-binding protein implicated in transcription termination	1.9
RSP_1949	*sufT*	FeS assembly SUF system protein	1.9
RSP_1924	*bioY_2*	Biotin transporter	1.9
RSP_1872	.	Glycosyl transferase, family 2	1.9
RSP_0577	.	Putative solute:sodium symporter small subunit	1.9
RSP_0519	*rfbE*	NAD-dependent epimerase/dehydratase	1.8
RSP_0986	*coaA*	Fructokinase	1.8
RSP_1327	*flgG_2*	Flagellar basal-body rod protein FlgF	1.8
RSP_0100	*ndhC_1*	NADH-quinone oxidoreductase subunit	1.8
RSP_0794	*oxyR_1*	Transcriptional regulator, LysR family	1.8
RSP_0072	.	Putative invasion protein	1.8
RSP_2414	.	Putative exonuclease, DNA ligase-associated	1.8
RSP_1121	*cat*	Acetyl transferase, Isoleucine patch superfamily	1.8
RSP_1782	.	Putative membrane protein	1.8
RSP_0522	*afr*	Putative oxidoreductase	1.8
RSP_1366	*arfB*	Protein chain release factor B	1.8
RSP_0788	.	Putative kinase involved in propanediol utilization	1.8

RSP_0310	*ureH*	Urease accessory protein UreD	1.8
RSP_2177	*dprA*	DNA protecting protein DprA	1.8
RSP_0338	*dagK*	Sphingosine/diacylglycerol kinase-like enzyme	1.8
RSP_2754	.	Uncharacterized protein	1.8
RSP_1910	.	Flp pilus assembly protein CpaB	1.8
RSP_2625	.	NifU-related protein involved in Fe-S cluster formation	1.8
RSP_0903	.	Catalase	1.8
RSP_1142	.	Putative membrane protein	1.8
RSP_0773	*uxuA*	Mannonate dehydratase	1.8
RSP_1302	*motB_3*	Putative chemotaxis MotB protein	1.8
RSP_1334	.	Putative chemotactic signal-response protein CheL	1.8
RSP_0117	*ctaD_1*	Cytochrome-c oxidase	1.8
RSP_1082	.	Formate dehydrogenase delta subunit	1.8
RSP_1286	*cmpR*	RuBisCO operon transcriptional regulator, CbbR	1.8
RSP_0381	.	Uncharacterized protein	1.8
RSP_1328	.	Flagellar biosynthetic protein FliQ	1.8
RSP_0018	*lysN*	Transcriptional regulator, gntR family	1.8
RSP_2351	.	Uncharacterized protein	1.8
RSP_1925	*gntR_1*	Transcriptional regulator, GntR family	1.8
RSP_1904	.	Flp pilus assembly protein TadB	1.8
RSP_2371	*fabG_3*	3-oxoacyl-(Acyl-carrier protein) reductase / Short-chain dehydrogenase/reductase SDR	1.7
RSP_0518	.	Putative glycosyltransferase protein	1.7
RSP_2213	*tsaB*	Universal bacterial protein YeaZ	1.7
RSP_1918	.	Putative alpha/beta hydrolase	1.7
RSP_2084	.	Uncharacterized protein	1.7
RSP_0058	.	FliK, flagellar hook-length control protein	1.7
RSP_1890	*yofA*	Transcriptional regulator, LysR family	1.7
RSP_0080	*flgE_1*	Flagellar hook protein FlgE	1.7
RSP_1888	.	Acetyltransferase, GNAT family	1.7
RSP_2984	*hemA*	5-aminolevulinate synthase 1	1.7
RSP_0114	.	Uncharacterized protein	1.7
RSP_1076	*sohB*	Peptidase family S49	1.7
RSP_6018	*sbmC*	DNA gyrase inhibitor	1.7
RSP_2591	*degU_4*	Two component transcriptional regulator, LuxR family	1.7
RSP_0589	.	Uncharacterized protein	1.7
RSP_0751	.	Uncharacterized protein	1.7
RSP_0527	*rpoN_1*	RNA polymerase sigma-54 factor	1.7
RSP_0660	.	Response regulator of the LytR/AlgR family	1.7
RSP_2664	.	Putative small integral membrane protein	1.7
RSP_0418	.	Uncharacterized protein	1.7
RSP_0999	.	UPF0301 protein RHOS4_26140	1.7

RSP_1326	*flgG_3*	Flagellar basal-body rod protein FlgG	1.7
RSP_1325	.	Flagella basal body P-ring formation protein FlgA	1.7
RSP_0042	*cheA_2*	Histidine kinase	1.7
RSP_2945	*ccmE*	Cytochrome c-type biogenesis protein CcmE	1.6
RSP_1788	*fruA*	PTS system protein D-fructose-specific IICB componenst (F1P-forming), Frc family	1.6
RSP_2028	.	SnoaL-like domain-containing protein	1.6
RSP_1591	*yqjG*	Putative Glutathione S-transferase	1.6
RSP_2881	.	Uncharacterized protein	1.6
RSP_2792	*prmA_2*	Putative monooxygenase alpha subunit	1.6
RSP_0528	*nifW2*	Nitrogenase-stabilizing/protective protein NifW	1.6
RSP_1308	.	DNA-binding protein with helix-turn-helix protein	1.6
RSP_1370	*malL*	Alpha amylase, catalytic subdomain protein	1.6
RSP_1880	.	Peptidoglycan-binding domain-containing protein	1.6
RSP_2026	*rhaS*	Transcriptional regulator, AraC family	1.6
RSP_0576	*mctC*	Na+/solute symporter	1.6
RSP_2878	*coxS*	Carbon-monoxide dehydrogenase small chain	1.6
RSP_1601	*ubiE_1*	Methylase involved in ubiquinone/menaquinone biosynthesis	1.6
RSP_2443	*srrA*	Putative Chemotaxis response regulator, CheY5	1.6
RSP_0795	.	YCII domain-containing protein	1.6
RSP_0034	*flhA_1*	Flagellar biosynthesis protein FlhA	1.6
RSP_0540	*nifD_1*	Nitrogenase protein alpha chain	1.6
RSP_2370	*ydjH*	Putative sugar kinase	1.6
RSP_1220	*hrcA*	Heat-inducible transcription repressor HrcA	1.6
RSP_0626	*infA*	Translation initiation factor IF-1	1.6
RSP_1081	*fdhD*	Sulfur carrier protein FdhD	1.6
RSP_1295	*acdA*	Putative acyl-CoA dehydrogenase	1.6
RSP_2720	*rhtC_1*	LysE/RhtB family amino acid efflux pump	1.6
RSP_2670	*glpD*	Glycerol-3-phosphate dehydrogenase	1.6
RSP_2944	.	Putative secretion activating protein	1.6
RSP_1997	*lexA*	LexA repressor	1.6
RSP_1781	.	Putative membrane-anchored oxidoreductase, similar to L-sorbosone dehydrogenase	1.6
RSP_1305	.	Putative flagellar hook-associated protein	1.6
RSP_2435	*cheW_2*	Chemotaxis scaffold protein, CheW1	1.6
RSP_2214	*nfuA*	Nitrogen-fixing NifU	1.6
RSP_1501	.	Anti-sigma factor antagonist	1.6
RSP_2224	*mdtA_3*	Multidrug efflux pump, membrane fusion protein (MFP) family	1.6
RSP_2024	*cspA_3*	Cold-shock DNA-binding protein family	1.6
RSP_0476	*fucA_1*	L-fuculose-phosphate aldolase	1.5
RSP_1787	*lacC*	Phosphofructokinase	1.5
RSP_0765	*tehB*	SAM-dependent methyltransferase	1.5

RSP_0301	*amiC_1*	Amino acid/amide ABC transporter substrate-binding protein, HAAT family	1.5
RSP_1647	*sppA*	Putative peptidase	1.5
RSP_0113	*ywiE*	Choline phosphatase	1.5
RSP_1985	.	Uncharacterized protein	1.5
RSP_1922	*gabR*	Transcriptional regulator, GntR family	1.5
RSP_0263	*lgoD_1*	2-desacetyl-2-hydroxyethyl bacteriochlorophyllide A dehydrogenase	1.5
RSP_0048	*cheR2*	MCP methyltransferase, CheR3	1.5
RSP_1581	.	Uncharacterized protein	1.5
RSP_2226	*glgX_3*	Glycosidase	1.5
RSP_2864	*rpe*	Ribulose-phosphate 3-epimerase	1.5
RSP_0044	*tap_1*	Putative cytoplasmic chemoreceptor, TlpT	1.5
RSP_1207	*hslO*	Putative Hsp33 protein	1.5
RSP_0284	.	2-vinyl bacteriochlorophyllide hydratase	1.5
RSP_2577	.	Isocytochrome c2	1.5
RSP_0752	*wecD*	Acetyltransferase (GNAT) family	1.5
RSP_0889	*glnB_1*	Nitrogen regulatory protein P-II	1.5
RSP_0049	*cheA_1*	Histidine kinase	1.5
RSP_2620	.	Uncharacterized protein	1.5
RSP_2440	*tsr*	Methyl accepting chemotaxis protein	1.5
RSP_1336	*flgD_2*	Basal-body rod modification protein FlgD	1.5
RSP_0615	.	Oxidoreductase	1.5
RSP_0559	*msrA*	Peptide methionine sulfoxide reductase MsrA	1.5
RSP_2413	*ligB*	DNA ligase (ATP)	1.5
RSP_0923	*map*	Methionine aminopeptidase	1.5
RSP_0166	*dksA_1*	Transcriptional regulator, TraR/DksA family	1.5
RSP_2882	*degU_3*	Two component transcriptional regulator, LuxR family	1.5
RSP_1400	*dinB*	DNA polymerase IV	1.5
RSP_0089	*yieH_2*	Haloacid dehalogenase superfamily protein, subfamily IA, variant 3 with third motif having DD or ED	1.5
RSP_1978	*dnaJ_1*	Molecular chaperone, DnaJ family	1.5
RSP_1303	*flgE_2*	Flagellar basal body protein	1.5
RSP_0370	.	TerB domain-containing protein	1.5
RSP_0546	*nifB_1*	FeMo cofactor biosynthesis protein NifB	1.5
RSP_2368	*rbsC_2*	Monosaccharide ABC transporter membrane protein, CUT2 family	1.5
RSP_0940	*ribY*	ABC transporter, periplasmic substrate binding protein	1.5
RSP_0115	.	Class I triheme cytochrome c	1.5
RSP_2667	*ugpC*	Carbohydrate ABC transporter ATP-binding protein, CUT1 family	1.5
RSP_2330	*leuA_2*	2-isopropylmalate synthase	1.5
RSP_1498	*hspQ*	Hemimethylated DNA binding domain protein	1.4
RSP_2433	*cheY_2*	Chemotaxis response regulator, CheY2	1.4
RSP_0472	.	Metal-dependent hydrolase	1.4

RSP_0171	*tcrX*	Response regulator receiver protein	1.4
RSP_1332	*fliI_2*	H(+)-transporting two-sector ATPase	1.4
RSP_2072	.	Phage-related Holin protein	1.4
RSP_1497	*lolA*	Outer membrane lipoprotein carrier protein	1.4
RSP_0574	.	Uncharacterized protein	1.4
RSP_1916	*nsaD*	2-hydroxychromene-2-carboxylate isomerase	1.4
RSP_2070	.	Phage-related terminase	1.4
RSP_0699	*hemN_1*	Oxygen-independent coproporphyrinogen III oxidase	1.4
RSP_1410	*msrP*	Protein-methionine-sulfoxide reductase catalytic subunit MsrP	1.4
RSP_2481	*cysE*	Serine acetyltransferase	1.4
RSP_2581	.	Rhodanese	1.4
RSP_1917	.	Cupin_2 domain-containing protein	1.4
RSP_0234	*tar*	TlpL, putative cytoplasmic chemoreceptor	1.4
RSP_2259	*yqaA*	Transmembrane protein	1.4
RSP_1072	*moaD*	Molybdopterin synthase subunit MoaD	1.4
RSP_0539	*nifK_1*	Nitrogenase molybdenum-iron protein beta chain	1.4
RSP_2410	*rpoH_2*	RNA polymerase sigma factor RpoH	1.4
RSP_0059	.	Flagellar protein FliL	1.4
RSP_0077	*flgH_1*	Flagellar L-ring protein	1.4
RSP_0361	*tdcG*	L-serine dehydratase	1.4
RSP_0262	*bchX*	Chlorophyllide reductase 35.5 kDa chain	1.4
RSP_0304	*ureE1*	Urease accessory protein UreE	1.4
RSP_2952	.	BolA-like protein	1.4
RSP_0408	.	Uncharacterized protein	1.4
RSP_2365	*xypA*	Monosaccharide ABC transporter substrate-binding protein, CUT2 family	1.4
RSP_0101	*nuoB_1*	NADH-quinone oxidoreductase subunit B 2	1.4
RSP_2360	.	Putative head portal protein, HK97 family	1.4
RSP_2372	*ald_2*	Aldehyde dehydrogenase (NAD-dependent)	1.3
RSP_1678	*recO*	DNA repair protein RecO	1.3
RSP_1393	*thiQ*	Thiamine import ATP-binding protein ThiQ	1.3
RSP_2930	.	Hint_2 domain-containing protein	1.3
RSP_0454	*cckA_1*	Histidine kinase	1.3
RSP_2323	.	Putative N-formylglutamate amidohydrolase	1.3
RSP_2619	*mnmA*	tRNA-specific 2-thiouridylase MnmA	1.3
RSP_2389	*gpx1*	Glutathione peroxidase	1.3
RSP_2367	*rbsC_1*	Monosaccharide ABC transporter membrane protein, CUT2 family	1.3
RSP_1194	*grxC*	Glutaredoxin	1.3
RSP_0904	*yfeA*	ABC Mn+2/Fe+2 transporter, periplasmic substrate-binding protein SitA	1.3
RSP_0619	.	Putative ester cyclase	1.3
RSP_0122	*anoR*	Transcriptional regulator, LuxR family	1.3

RSP_0865	*rsfS*	Ribosomal silencing factor RsfS	1.3
RSP_2649	*loiP_1*	Zn-dependent protease	1.3
RSP_0317	*hemN_2*	Coproporphyrinogen III oxidase, anaerobic 1	1.3
RSP_0487	.	TRAP-T family transporter, DctP (Periplasmic binding) subunit	1.3
RSP_0590	.	Uncharacterized protein	1.3
RSP_1852	*folE2*	GTP cyclohydrolase FolE2	1.3
RSP_1903	.	Flp pilus assembly protein TadC	1.3
RSP_1025	.	Uncharacterized protein	1.3
RSP_2572	*fixK_2*	CrpK, Fnr-type transcriptional regulator	1.3
RSP_0351	*pld1*	D-threo-aldose 1-dehydrogenase	1.3
RSP_0880	*thiO*	Putative oxidoreductase, possibly D-amino acid oxidase protein	1.3
RSP_0281	*bchE*	Anaerobic magnesium-protoporphyrin IX monomethyl ester cyclase	1.3
RSP_0303	*ureF*	Urease accessory protein UreF	1.3
RSP_1316	*motA*	Flagellar motor protein	1.3
RSP_1163	*nusA*	Transcription termination/antitermination protein NusA	1.3
RSP_1577	*lrp_3*	Transcriptional regulator, AsnC family	1.3
RSP_1606	*glcC*	Transcriptional regulator, GntR family	1.3
RSP_2430	.	Uncharacterized protein	1.3
RSP_1382	*rlmN*	Dual-specificity RNA methyltransferase RlmN	1.3
RSP_2153	*lpxH*	Metallo-phosphoesterase	1.3
RSP_1335	.	Flagellar hook-length control protein FliK	1.3
RSP_0941	*cmpD_2*	ABC transporter, ATPase subunit	1.2
RSP_0316	*degU_1*	Transcriptional regulator, LuxR family	1.2
RSP_2366	*rbsA_2*	Ribose import ATP-binding protein RbsA	1.2
RSP_1956	.	Uncharacterized protein	1.2
RSP_2771	*gppA*	Putative Exopolyphosphatase	1.2
RSP_0754	.	Uncharacterized protein	1.2
RSP_0170	.	Uncharacterized protein	1.2
RSP_0685	*rlmG*	16S rRNA m(2)G 1207 methyltransferase	1.2
RSP_2592	.	Uncharacterized protein	1.2
RSP_1470	*rnhB*	Ribonuclease HII	1.2
RSP_0625	*yhdE*	dTTP/UTP pyrophosphatase	1.2
RSP_2294	*gloB*	Hydroxyacylglutathione hydrolase	1.2
RSP_1162	*rimP*	Ribosome maturation factor RimP	1.2
RSP_1923	*bioB*	Biotin synthase	1.2
RSP_1357	*barA*	Histidine kinase	1.2
RSP_1245	*iolG*	Putative oxidoreductase, Gfo/Idh/MocA family	1.2
RSP_0659	.	Putative phosphoesterase	1.2
RSP_1996	*comEC*	Competence protein	1.2
RSP_2220	*fliG_2*	Flagellar motor switch protein FliG	1.2

RSP_1821	*ydeP*	Molybdopterin-containing oxidoreductase, putative formate dehydrogenase	1.2
RSP_2722	*cysS*	Cysteine--tRNA ligase	1.2
RSP_1584	*cheA_4*	Histidine kinase	1.2
RSP_2709	*dxr*	1-deoxy-D-xylulose 5-phosphate reductoisomerase	1.2
RSP_2658	*erpA*	HesB/YadR/YfhF family protein	1.2
RSP_6056	*anmK*	Anhydro-N-acetylmuramic acid kinase	1.2
RSP_7566	.	Putative small protein	1.2
RSP_1948	*sufA*	Iron-sulfur cluster assembly accessory protein	1.2
RSP_1092	*rpoE*	ECF RNA polymerase sigma factor RpoE	1.2
RSP_1889	*ybhL*	FtsH-interacting integral membrane protein	1.2
RSP_2933	.	Formiminoglutamate deiminase	1.2
RSP_0616	*nimR*	Transcriptional regulator, AraC family	1.2
RSP_0474	*cycP*	Cytochrome c'	1.2
RSP_0300	*livH_1*	Amino acid/amide ABC transporter membrane protein 1, HAAT family	1.2
RSP_0443	*iscR*	Transcriptional regulator, BadM/Rrf2 family	1.2
RSP_0294	.	Aerobic magnesium-protoporphyrin IX monomethyl ester [oxidative] cyclase	1.2
RSP_0514	*strE*	dTDP-glucose 4,6-dehydratase protein	1.2
RSP_2589	*gloC_2*	Zn-dependent hydrolase, glyoxylase	1.2
RSP_0638	*puuB_1*	Putative FAD dependent oxidoreductase protein	1.2
RSP_6073	.	Uncharacterized protein	1.2
RSP_2558	*hprK_1*	Hpr(Ser) kinase/phosphatase	1.2
RSP_1012	*davD*	Succinate semialdehyde dehydrogenase	1.2
RSP_2503	*glmU*	Bifunctional protein GlmU	1.2
RSP_0892	*prsD_1*	ABC protein toxin exporter, fused ATPase and inner membrane domain protein	1.1
RSP_2152	.	Putative ceramide glucosyltransferase	1.1
RSP_1531	*hslV*	ATP-dependent protease subunit HslV	1.1
RSP_1529	*trxA*	Thioredoxin	1.1
RSP_2136	.	Uncharacterized protein	1.1
RSP_2665	*sugB_2*	Carbohydrate ABC transporter membrane protein 2, CUT1 family	1.1
RSP_1307	*flgI_2*	Flagellar P-ring protein 2	1.1
RSP_1321	.	Flagellar biosynthesis pathway, component FliR	1.1
RSP_1219	*grpE*	Protein GrpE	1.1
RSP_0893	*prsE_1*	Membrane fusion protein (MFP) family protein	1.1
RSP_0717	.	RpsU-divergently transcribed protein	1.1
RSP_0244	*puuD*	Putative glutamine amidotransferase	1.1
RSP_0674	*dnaX_2*	DNA polymerase III, delta prime subunit	1.1
RSP_0285	*bchN*	Light-independent protochlorophyllide reductase subunit N	1.1
RSP_0070	*fliD*	Flagellar hook-associated protein 2	1.1
RSP_1195	*pyrE_2*	Competence protein F	1.1

RSP_1536	*phaJ_2*	Acyl dehydratase	1.1
RSP_0715	.	Ribonuclease T2	1.1
RSP_1456	*thiK*	Phosphotransferase family protein	1.1
RSP_1742	*degP_2*	Periplasmic serine endoprotease DegP-like	1.1
RSP_1120	*prsE_2*	Membrane fusion protein (MFP) family protein	1.1
RSP_0280	.	Bacteriochlorophyll synthase 23 kDa chain	1.1
RSP_2377	*tdh*	L-threonine 3-dehydrogenase	1.1
RSP_1667	.	NYN domain-containing protein	1.1
RSP_2391	*thpR*	RNA 2',3'-cyclic phosphodiesterase	1.1
RSP_2621	*ctrA*	Two component transcriptional regulator, winged helix family	1.1
RSP_1544	.	Putative periplasmic lipoprotein	1.1
RSP_0595	*blc*	Outer membrane lipoprotein Blc	1.1
RSP_2122	*metH_2*	Dimethylamine corrinoid protein	1.1
RSP_1952	*cspA_4*	Cold-shock DNA-binding protein family	1.1
RSP_1309	*fliP_2*	Flagellar biosynthetic protein FliP	1.1
RSP_1592	*acsA_2*	Propionyl-CoA synthetase	1.1
RSP_1324	*flgH_2*	Flagellar L-ring protein	1.1
RSP_0308	*ureB*	Urease subunit beta	1.0
RSP_0847	*walR*	Two component transcriptional regulator, winged helix family	1.0
RSP_0760	.	Transcriptional regulator, MarR family	1.0
RSP_6024	.	Putative conserved small protein	1.0
RSP_2029	*fabG_4*	Short chain dehydrogenase	1.0
RSP_2721	.	Uncharacterized protein	1.0
RSP_0231	*motB_1*	Flagellar MotB protein	1.0
RSP_2785	*cycF*	Cytochrome c-554	1.0
RSP_0102	*nuoC*	NADH-quinone oxidoreductase subunit C/D	1.0
RSP_2022	*hupC_2*	Cytochrome b/diheme cytochrome c hybrid protein	1.0
RSP_2666	*sugA_2*	Carbohydrate ABC transporter membrane protein 1, CUT1 family	1.0
RSP_2218	*erfK_1*	YkuD domain-containing protein	1.0
RSP_2388	.	NTP pyrophosphohydrolase	1.0
RSP_0427	.	Uncharacterized protein	1.0
RSP_2501	*nadC*	Nicotinate-nucleotide pyrophosphorylase	1.0
RSP_0152	.	P-loop ATPase	1.0
RSP_2799	*adh_2*	Zinc-containing alcohol dehydrogenase	1.0
RSP_1574	.	Cytochrome b562	1.0
RSP_1649	.	Putative transporter, DMT superfamily	1.0
RSP_1384	.	Asparaginase	-1.0
RSP_1707	*tuf1_1*	Elongation factor Tu	-1.0
RSP_2971	*queA*	S-adenosylmethionine:tRNA ribosyltransferase-isomerase	-1.0
RSP_0810	.	Uncharacterized protein involved in cysteine biosynthesis	-1.0
RSP_1897	.	Magnesium transporter MgtE	-1.0

RSP_0358	*garB*	Glutathione reductase	-1.0
RSP_2428	*cobU*	Nicotinate-nucleotide--dimethylbenzimidazole phosphoribosyltransferase	-1.0
RSP_2969	.	Uncharacterized protein	-1.0
RSP_0411	.	Uncharacterized protein	-1.0
RSP_1479	*oppC*	ABC oligopeptide transporter, inner membrane subunit OppC	-1.0
RSP_0437	*sufC*	Suf C, ATPase	-1.0
RSP_0796	*cobQ*	Cobyric acid synthase	-1.0
RSP_0344	*rbsA_1*	Nucleoside ABC transporter ATP-binding protein	-1.0
RSP_2522	*nuoH_2*	NADH-quinone oxidoreductase subunit H 1	-1.0
RSP_1830	.	SURF1-like protein	-1.0
RSP_2738	*cysA1*	Putative Rhodanese-related sulfurtransferase	-1.0
RSP_2507	*ompW*	Outer membrane protein	-1.0
RSP_0551	*tmk*	Thymidylate kinase	-1.0
RSP_2109	.	Uncharacterized protein	-1.0
RSP_2328	*mreC*	Cell shape protein MreC	-1.0
RSP_0425	*metG*	Methionine--tRNA ligase	-1.0
RSP_2345	*pdxH*	Pyridoxine/pyridoxamine 5'-phosphate oxidase	-1.0
RSP_1467	*alkB2*	Alkane 1-monooxygenase	-1.0
RSP_1030	*scrK_2*	Putative pfkB family carbohydrate kinase	-1.0
RSP_2301	*prs*	Ribose-phosphate pyrophosphokinase	-1.0
RSP_2708	*cdsA*	Phosphatidate cytidylyltransferase	-1.0
RSP_1833	*ydaF*	Putative ribosomal-protein-alanine acetyltransferase	-1.0
RSP_0745	*phaA*	Acetyl-CoA acetyltransferase	-1.0
RSP_2047	.	ThiF family protein	-1.1
RHOS4_24670	.	UPF0178 protein RHOS4_24670	-1.1
RSP_0007	.	Putative outer membrane protein	-1.1
RSP_6009	.	Uncharacterized protein	-1.1
RSP_2647	*rlmI*	SAM-dependent methyltransferase	-1.1
RSP_0191	*accC*	Biotin carboxylase	-1.1
RSP_0021	*rpsI*	30S ribosomal protein S9	-1.1
RSP_1602	*dctM_6*	TRAP-T family transporter, DctM (12TMs) subunit	-1.1
RSP_1842	.	Putative transporter, DME family, DMT superfamily	-1.1
RSP_1287	*livF_4*	Amino acid/amide ABC transporter ATP-binding protein 2, HAAT family	-1.1
RSP_2534	*rnj*	Putative hydrolase of the metallo-beta-lactamase superfamily	-1.1
RSP_1675	*rnc*	Ribonuclease 3	-1.1
RSP_1523	*amgK*	Aminoglycoside phosphotransferase	-1.1
RSP_1154	.	Uncharacterized protein	-1.1
RSP_0477	.	Putative hydrolase of the alpha/beta-hydrolase fold protein	-1.1
RSP_0825	*nadK*	NAD kinase	-1.1
RSP_1727	*rplE*	50S ribosomal protein L5	-1.1

RSP_0755	*nusB*	Transcription antitermination protein NusB	-1.1
RSP_0161	.	Spermidine/putrescine-binding periplasmic protein	-1.1
RSP_1795	*serS*	Serine--tRNA ligase	-1.1
RSP_2773	.	Uncharacterized protein	-1.1
RSP_1726	*rplX*	50S ribosomal protein L24	-1.1
RSP_2307	*ppaC*	Pyrophosphate phospho-hydrolase	-1.1
RSP_0962	*lpd3*	Dihydrolipoyl dehydrogenase	-1.1
RSP_0852	*fghA*	S-formylglutathione hydrolase	-1.1
RSP_1913	*scoB*	3-oxoadipate CoA-transferase, beta subunit	-1.1
RSP_2211	*tmpC*	Nucleoside-binding protein	-1.1
RSP_2903	*surA*	Parvulin-like PPIase	-1.1
RSP_2684	*yrrK*	Putative pre-16S rRNA nuclease	-1.1
RSP_6153	.	Putative nucleotide kinase	-1.1
RSP_0882	*dapA*	4-hydroxy-tetrahydrodipicolinate synthase	-1.1
RSP_1109	*dcsD*	Cysteine synthase	-1.1
RSP_1861	.	Putative dihydroneopterin aldolase	-1.1
RSP_0974	*sdhC*	Succinate dehydrogenase cytochrome b556 subunit	-1.1
RSP_2854	*macA*	Cation/multidrug efflux pump, Membrane fusion protein (MFP) family	-1.1
RSP_1001	*udg_2*	Type-4 uracil-DNA glycosylase	-1.1
RSP_0140	*rpsR*	30S ribosomal protein S18	-1.1
RSP_2015	.	Arginine/ornithine transport system ATPase	-1.1
RSP_0693	*ccoP*	Cbb3-type cytochrome c oxidase subunit CcoP	-1.1
RSP_2735	*pgl*	6-phosphogluconolactonase	-1.1
RSP_2249	*tetA*	Multidrug (Tetracycline) efflux pump, Major facilitator superfamily (MFS)	-1.1
RSP_2283	*rpsD*	30S ribosomal protein S4	-1.1
RSP_1800	.	Uncharacterized protein	-1.1
RSP_0347	*cysW_1*	ABC transporter, inner membrane subunit	-1.1
RSP_2912	*mazG*	Putative pyrophosphatase	-1.1
RSP_2134	*dsbD*	Putative cytochrome c-type biogenesis protein CcdA	-1.1
RSP_1735	*secY*	Protein translocase subunit SecY	-1.1
RSP_1596	*deoB*	Phosphopentomutase	-1.1
RSP_2541	*tatC*	Sec-independent protein translocase protein TatC	-1.1
RSP_2980	*aspC*	Aminotransferase	-1.1
RSP_1048	*rpsP*	30S ribosomal protein S16	-1.1
RSP_2333	*nadE_1*	Glutamine-dependent NAD(+) synthetase	-1.1
RSP_0781	*ppk*	Polyphosphate kinase	-1.1
RSP_2715	*lpxI*	Uncharacterized protein	-1.1
RSP_0158	*bcsA*	Cellulose synthase-like protein	-1.1
RSP_0571	*truA*	tRNA pseudouridine synthase A	-1.1
RSP_0321	.	NorD Nitric oxide reductase activation protein	-1.1

RSP_1595	*deoA*	Thymidine phosphorylase	-1.1
RSP_0449	*egtC*	Putative glutamine amidotransferase	-1.1
RSP_2306	*yutF*	Putative HAD superfamily protein	-1.1
RSP_2894	*ndk*	Nucleoside diphosphate kinase	-1.1
RSP_2607	*ntaB*	Nitrilotriacetate monooxygenase component B	-1.1
RSP_0392	*gloA*	Putative lactoylglutathione lyase	-1.2
RSP_1496	.	Uncharacterized protein	-1.2
RSP_2258	*purC_1*	Phosphoribosylaminoimidazole-succinocarboxamide synthase	-1.2
RSP_2867	*rpoN_2*	RNA polymerase sigma-54 factor	-1.2
RSP_0463	*fieF*	Cation efflux transporter, CDF family	-1.2
RSP_0322	*nirQ*	NorQ protein required for nitric oxide reductase activity	-1.2
RSP_2737	*ridA*	SsRNA endoribonuclease L-PSP	-1.2
RSP_1201	.	YkuD domain-containing protein	-1.2
RSP_1391	*thiB*	Thiamine-binding periplasmic protein	-1.2
RSP_6008	*ftsN*	Sporulation and cell division-related protein	-1.2
RSP_2407	*luxQ_2*	Histidine kinase	-1.2
RSP_2650	.	Uncharacterized protein	-1.2
RSP_4044	*pgk*	Phosphoglycerate kinase	-1.2
RSP_0720	.	CDP-diacylglycerol--serine O-phosphatidyltransferase	-1.2
RSP_2702	*dppB_2*	ABC oligo/dipeptide transporter, inner membrane subunit	-1.2
RSP_6204	.	Outer membrane protein	-1.2
RSP_2210	*rbsA_3*	Nucleoside ABC transporter ATP-binding protein	-1.2
RSP_0713	.	OpgC protein	-1.2
RSP_0725	*tlpA*	Thioredoxin, thioldisulfide interchange protein	-1.2
RSP_2909	*speB*	Agmatinase	-1.2
RSP_2004	*trpE*	Anthranilate synthase component 1	-1.2
RSP_1773	*sugE*	Multidrug efflux pump, SMR family, DMT superfamily	-1.2
RSP_2609	*dcd*	2'-deoxycytidine 5'-triphosphate deaminase	-1.2
RSP_2256	*dtd*	D-aminoacyl-tRNA deacylase	-1.2
RSP_0141	*rplI*	50S ribosomal protein L9	-1.2
RSP_1674	*lepB*	Signal peptidase I	-1.2
RSP_1039	*atpI*	ATP synthase protein I	-1.2
RSP_0934	*gpmI*	2,3-bisphosphoglycerate-independent phosphoglycerate mutase	-1.2
RSP_1478	*yejF_2*	ABC oligopeptide transporter, fused ATPase subunits	-1.2
RSP_1994	*gltA*	Citrate synthase	-1.2
RSP_1160	*kdsB*	3-deoxy-manno-octulosonate cytidylyltransferase	-1.2
RSP_1379	*pepA_2*	Cytosol aminopeptidase	-1.2
RSP_1854	*trkI_1*	Trk system potassium uptake protein	-1.2
RSP_1725	*rplN*	50S ribosomal protein L14	-1.2
RSP_0239	*pntB*	NAD(P) transhydrogenase subunit beta	-1.2
RSP_1729	*rpsH*	30S ribosomal protein S8	-1.2

RSP_1101	.	Uncharacterized protein	-1.2
RSP_2623	*recG*	ATP-dependent DNA helicase RecG	-1.2
RSP_0654	*ribN_3*	Putative transporter, DMT superfamily	-1.2
RSP_0676	.	PhnP-like protein	-1.2
RSP_0242	*kynU*	Kynureninase	-1.2
RSP_2917	*metC*	Putative cystathionine gamma-synthase beta-lyase	-1.2
RSP_1524	*murU*	Nucleotidyltransferase family protein	-1.2
RSP_2103	*murD*	UDP-N-acetylmuramoylalanine--D-glutamate ligase	-1.2
RSP_1802	*ccmB*	Heme exporter protein B	-1.2
RSP_0349	*cysA_1*	ABC transporter, ATPase subunit	-1.2
RSP_2113	*ftsA*	Cell division protein FtsA	-1.2
RSP_0957	*pyrD*	Dihydroorotate dehydrogenase (quinone)	-1.2
RSP_1969	*purM*	Phosphoribosylformylglycinamidine cyclo-ligase	-1.2
RSP_0150	*cph1*	Histidine kinase	-1.2
RSP_0352	*fdxE*	Putative ferredoxin	-1.2
RSP_0343	.	Nucleoside ABC transporter membrane protein	-1.2
RSP_0587	*ettA*	Energy-dependent translational throttle protein EttA	-1.2
RSP_1494	*dapL*	Putative aspartate aminotransferase	-1.2
RSP_1127	.	Acetyltransferase, GNAT family	-1.2
RSP_0646	.	ABC transporter, substrate-binding protein	-1.2
RSP_1723	*rplP*	50S ribosomal protein L16	-1.2
RSP_2327	.	Uncharacterized protein	-1.2
RSP_2716	*lpxB*	Lipid-A-disaccharide synthase	-1.2
RSP_2728	*estB*	Phospholipase/Carboxylesterase	-1.2
RSP_2332	.	Uncharacterized protein	-1.2
RSP_2698	*bamB*	Putative quinoprotein	-1.2
RSP_2250	*pytH*	Putative hydrolase or acyltransferase of alpha/beta superfamily	-1.2
RSP_2409	*rluD*	Pseudouridine synthase	-1.3
RSP_2810	*mkl_1*	ABC transporter, ATPase subunit	-1.3
RSP_1480	*oppB*	ABC oligopeptide transporter, inner membrane subunit OppB	-1.3
RSP_0971	.	Putative membrane protein	-1.3
RSP_2972	.	AsmA_2 domain-containing protein	-1.3
RSP_4041	*tyrS*	Tyrosine--tRNA ligase	-1.3
RSP_2395	*ccpA*	BCCP, cytochrome c peroxidase	-1.3
RSP_1646	.	Probable membrane transporter protein	-1.3
RSP_2910	*hipO*	Putative Metal-dependent amidase/aminoacylase/carboxypeptidase	-1.3
RSP_0431	*sufS_1*	Cysteine desulfurase	-1.3
RSP_1754	*erfK_2*	YkuD domain-containing protein	-1.3
RSP_1597	*add2*	Adenosine deaminase	-1.3
RSP_2974	*mepM*	Metalopeptidase	-1.3
RSP_2842	*trkG*	Potassium uptake transporter, transmembrane subunit, TrkH	-1.3

RSP_0651	*suhB_1*	Inositol monophosphatase protein	-1.3
RSP_2612	*fabH*	3-oxoacyl-[acyl-carrier-protein] synthase 3	-1.3
RSP_1461	*waaA_2*	3-deoxy-D-manno-octulosonic acid transferase	-1.3
RSP_0858	*ribN_2*	Transporter, RhaT family, DMT superfamily	-1.3
RSP_1797	*yajC*	Sec translocon accessory complex subunit YajC	-1.3
RSP_1360	.	YGGT family protein	-1.3
RSP_0708	*nudC*	NAD(+) diphosphatase	-1.3
RSP_0759	.	Putative capsule polysaccharide exporter	-1.3
RSP_1520	*regB*	Sensor histidine kinase RegB	-1.3
RSP_1980	*glcB*	Malate synthase G	-1.3
RSP_0895	*glpQ*	Phosphodiesterase	-1.3
RSP_0006	*ribN_5*	Putative transporter, DMT superfamily	-1.3
RSP_1183	*tamB*	Uncharacterized protein	-1.3
RSP_0656	.	Putative sodium/sulfate symporter	-1.3
RSP_2704	*miaA*	tRNA dimethylallyltransferase	-1.3
RSP_2200	*zntR_2*	Transcriptional regulator, MerR family	-1.3
RSP_2252	*thadh*	Threonine dehydratase	-1.3
RSP_2705	*pyrH*	Uridylate kinase	-1.3
RSP_2841	*trkA*	Trk system potassium uptake protein TrkA	-1.3
RSP_1709	*rpsG*	30S ribosomal protein S7	-1.3
RSP_0972	.	Putative conserved small protein	-1.3
RSP_2855	*mdtC_2*	Cation/multidrug efflux pump, RND superfamily	-1.3
RSP_1719	*rplB*	50S ribosomal protein L2	-1.3
RSP_2464	*fabF_2*	3-oxoacyl-[acyl-carrier-protein] synthase 2	-1.3
RSP_0844	*yggS*	Pyridoxal phosphate homeostasis protein	-1.3
RSP_0670	.	Cell division and transport-associated protein TolA	-1.3
RSP_1697	.	Uncharacterized protein	-1.3
RSP_2942	.	Sporulation and cell division-related protein	-1.3
RSP_1359	*recQ_3*	DNA helicase	-1.3
RSP_1365	*yhhQ*	Probable queuosine precursor transporter	-1.3
RSP_2254	.	ABC efflux transporter, fused ATPase and inner membrane subunits	-1.3
RSP_2216	*ilvE_1*	Probable branched-chain-amino-acid aminotransferase	-1.3
RSP_0154	*mmsB*	3-hydroxyisobutyrate dehydrogenase	-1.3
RSP_0187	*preA*	Dihydrothymine dehydrogenase	-1.3
RSP_1986	*gatB*	Aspartyl/glutamyl-tRNA(Asn/Gln) amidotransferase subunit B	-1.3
RSP_1166	*nudG*	NTP pyrophosphohydrolase	-1.3
RSP_2289	*motB_2*	Flagellar motor protein	-1.3
RSP_0776	*lolD*	Lipoprotein-releasing system ATP-binding protein LolD	-1.3
RSP_0671	*exbD_1*	Cell division and transport-associated protein TolR	-1.3
RSP_0139	*rpsF*	30S ribosomal protein S6	-1.3
RSP_0643	*pdtaS_1*	Periplasmic sensor signal transduction histidine kinase	-1.3

RSP_1221	*rph*	Ribonuclease PH	-1.3
RSP_1728	*rpsN*	30S ribosomal protein S14	-1.3
RSP_1702	*rplA*	50S ribosomal protein L1	-1.3
RSP_1701	*rplJ*	50S ribosomal protein L10	-1.3
RSP_0930	*folC*	Dihydrofolate synthase/folylpolyglutamate synthase	-1.3
RSP_2545	*surE*	5'-nucleotidase SurE	-1.4
RSP_0777	*lolC*	ABC lipoprotein efflux transporter, inner membrane subunit, LolE	-1.4
RSP_2106	*ftsW*	Cell wall polymerase	-1.4
RSP_1722	*rpsC*	30S ribosomal protein S3	-1.4
RSP_0385	*thrS*	Threonine--tRNA ligase	-1.4
RSP_0256	*pufM*	Reaction center protein M chain	-1.4
RSP_0748	*yfiC*	N-6 Adenine-specific DNA methylase	-1.4
RSP_1042	*soj_2*	ATPase involved in chromosome partitioning	-1.4
RSP_0933	.	Membrane-bound metallopeptidase	-1.4
RSP_2943	*argS*	Arginine--tRNA ligase	-1.4
RSP_1721	*rplV*	50S ribosomal protein L22	-1.4
RSP_0841	.	Uncharacterized protein	-1.4
RSP_1440	*fhuA*	TonB dependent, hydroxamate-type ferrisiderophore, outer membrane receptor	-1.4
RSP_1150	*uppP*	Undecaprenyl-diphosphatase	-1.4
RSP_0692	.	Protein RdxB	-1.4
RSP_1761	*pheT*	Phenylalanine--tRNA ligase beta subunit	-1.4
RSP_1005	*egtA*	Glutamate--cysteine ligase	-1.4
RSP_2734	*zwf*	Glucose-6-phosphate 1-dehydrogenase	-1.4
RSP_2431	*mgl*	O-acetylhomoserine sulfhydrylase	-1.4
RSP_0652	*galE_1*	UDP-glucose 4-epimerase	-1.4
RSP_0147	*glnA*	Glutamine synthetase	-1.4
RSP_0996	*mrpF*	Multisubunit potassium/proton antiporter, PhaF subunit	-1.4
RSP_0246	*msbA_2*	ABC lipid efflux transporter, fused ATPase and inner membrane subunits	-1.4
RSP_4045	*fbaB*	Fructose-bisphosphate aldolase	-1.4
RSP_0405	*recJ*	Putative single-stranded DNA-specific exonuclease	-1.4
RSP_2900	.	Lipopolysaccharide export system permease protein LptF	-1.4
RSP_2237	*emrA*	Membrane Fusion Protein (MFP) Family protein	-1.4
RSP_2941	*nagZ*	Glycoside hydrolase	-1.4
RSP_2300	*atpC*	ATP synthase epsilon chain 1	-1.4
RSP_2229	.	Chemotaxis multidomain, CheB methylesterase/CheR, Methylase	-1.4
RSP_1254	*ackA*	Acetate kinase	-1.4
RSP_0757	*ribBA*	3,4-dihydroxy-2-butanone 4-phosphate synthase	-1.4
RSP_1569	*hemL*	Glutamate-1-semialdehyde 2,1-aminomutase	-1.4
RSP_0375	*puuA_2*	Glutamate--putrescine ligase	-1.4
RSP_1995	*gltX*	Glutamate--tRNA ligase 1	-1.4

RSP_0979	*sdhB*	Succinate dehydrogenase iron-sulfur subunit	-1.4
RSP_1810	*murJ*	Probable lipid II flippase MurJ	-1.4
RSP_0831	*trpA*	Tryptophan synthase alpha chain	-1.4
RSP_0377	*puuA_1*	L-glutamine synthetase	-1.4
RSP_1462	*lpxK*	Tetraacyldisaccharide 4'-kinase	-1.4
RSP_0433	.	Yip1 domain-containing protein	-1.4
RSP_1460	.	Uncharacterized protein	-1.4
RSP_1598	*upp*	Uracil phosphoribosyltransferase	-1.4
RSP_1096	.	Putative zinc protease	-1.4
RSP_2091	*ydcO*	Benzoate transporter, BenE	-1.4
RSP_0479	*efp_1*	Elongation factor P	-1.4
RSP_1839	.	Putative acetyltransferase, GNAT family	-1.4
RSP_0434	*sufD*	SufD protein	-1.5
RSP_0129	*metN*	Methionine import ATP-binding protein MetN	-1.5
RSP_0432	.	Yip1 domain-containing protein	-1.5
RSP_1089	.	Sugar/cation symporter, GPH family	-1.5
RSP_1703	*rplK*	50S ribosomal protein L11	-1.5
RSP_0815	*aspS*	Aspartate--tRNA(Asp/Asn) ligase	-1.5
RSP_1570	*purK*	N5-carboxyaminoimidazole ribonucleotide synthase	-1.5
RSP_2914	*rihA*	Nucleoside hydrolase	-1.5
RSP_0829	*lldD*	Lactate dehydrogenase	-1.5
RSP_2956	*tktA*	Transketolase	-1.5
RSP_0201	*ynjC*	ABC transporter, fused inner membrane subunits	-1.5
RSP_2902	*lptD*	LPS-assembly protein LptD	-1.5
RSP_2209	.	Nucleoside ABC transporter membrane protein	-1.5
RSP_2270	*gatA*	Glutamyl-tRNA(Gln) amidotransferase subunit A	-1.5
RSP_2904	*pdxA*	4-hydroxythreonine-4-phosphate dehydrogenase	-1.5
RSP_1927	.	Probable membrane transporter protein	-1.5
RSP_0830	*guaB_1*	CBS-domain-containing membrane protein	-1.5
RSP_2711	*bamA*	Outer membrane protein assembly factor BamA	-1.5
RSP_0742	*dmdC_1*	Putative acyl-CoA dehydrogenase	-1.5
RSP_2645	*eda*	2-dehydro-3-deoxy-phosphogluconate aldolase	-1.5
RSP_2098	*ftsI*	Cell division protein FtsI/penicillin-binding protein 2	-1.5
RSP_2158	*spuE*	ABC transporter, periplasmic solute-binding protein	-1.5
RSP_1069	.	4Fe-4S ferredoxin-type domain-containing protein	-1.5
RSP_0887	*pbpG*	Penicillin-insensitive transglycosylase	-1.5
RSP_0404	*glpX*	Fructose-1,6-bisphosphatase	-1.5
RSP_1291	*amiC_2*	Amino acid/amide ABC transporter substrate-binding protein, HAAT family	-1.5
RSP_2458	*mkl_2*	ABC transporter, ATPase subunit	-1.5
RSP_1694	*bdhA_2*	Putative short-chain dehydrogenase/reductase	-1.5
RSP_2257	*scrK_1*	Putative Fructokinase	-1.5

RSP_1468	*dpnA*	Methyltransferase	-1.5
RSP_1856	*glyQ*	Glycine--tRNA ligase alpha subunit	-1.5
RSP_0024	*rfbC*	dTDP-4-dehydrorhamnose 3,5-epimerase	-1.5
RSP_1068	*argB*	Acetylglutamate kinase	-1.5
RSP_0836	.	Flavoprotein	-1.5
RSP_1463	*uctC*	Acyl-CoA transferase/carnitine dehydratase	-1.5
RSP_0650	.	Putative transglycosylase	-1.5
RSP_0997	*mrpG*	Multisubunit potassium/proton antiporter, PhaG subunit	-1.5
RSP_2020	.	DHC, diheme cytochrome c	-1.5
RSP_0706	*pheA*	Prephenate dehydratase	-1.5
RSP_1187	.	Putative lipoprotein DUF940	-1.5
RSP_2739	.	Sulf_transp domain-containing protein	-1.5
RSP_0311	*thrB*	Aminotransferase protein	-1.5
RSP_0455	*rsmB_1*	Putative NOL1/NOP2/sun family protein	-1.5
RSP_0854	.	Ornithine cyclodeaminase	-1.5
RSP_2811	.	ABC transporter, periplasmic substrate-binding protein	-1.5
RSP_2901	*lptG*	Putative permease	-1.5
RSP_2465	*mltG*	Endolytic murein transglycosylase	-1.5
RSP_2204	*dinF*	Probable multidrug resistance protein NorM	-1.5
RSP_1763	*pheS*	Phenylalanine--tRNA ligase alpha subunit	-1.5
RSP_2736	*pgi*	Glucose-6-phosphate isomerase	-1.5
RSP_0029	*cat1*	Acetyl-CoA hydrolase/transferase family protein	-1.6
RSP_0015	*potA_1*	Spermidine/putrescine import ATP-binding protein PotA	-1.6
RSP_2896	.	UPF0056 inner membrane protein	-1.6
RSP_1525	.	Putative Helicase/Exonuclease	-1.6
RSP_1720	*rpsS*	30S ribosomal protein S19	-1.6
RSP_0312	*gabT*	Aminotransferase class-III	-1.6
RSP_0677	.	Malonate transporter, mdcF, AEC family	-1.6
RSP_1065	.	Putative Fe-S protein	-1.6
RSP_0341	*codA*	Cytosine deaminase	-1.6
RSP_1167	*argJ*	Arginine biosynthesis bifunctional protein ArgJ	-1.6
RSP_1306	.	Histidine kinase	-1.6
RSP_0566	.	Putative transporter, DMT superfamily	-1.6
RSP_2394	*waaA_1*	3-deoxy-D-manno-octulosonic acid transferase	-1.6
RSP_0814	.	Acetyltransferase	-1.6
RSP_0156	*acrC*	Acyl-CoA dehydrogenase	-1.6
RSP_1766	*ttuE_2*	Pyruvate kinase	-1.6
RSP_1180	*xylF_2*	Xylose-binding protein	-1.6
RSP_0403	*hom*	Homoserine dehydrogenase	-1.6
RSP_0342	.	Nucleoside ABC transporter membrane protein	-1.6
RSP_0653	*rkpK*	UDP-glucose 6-dehydrogenase	-1.6

RSP_2253	.	Putative hydrolase or acyltransferase	-1.6
RSP_2968	*lpdC*	Dihydrolipoyl dehydrogenase	-1.6
RSP_1929	*pyrE_1*	Orotate phosphoribosyltransferase	-1.6
RSP_2299	*atpD*	ATP synthase subunit beta 1	-1.6
RSP_0842	.	Putative porin	-1.6
RSP_0016	*ydcV_1*	ABC spermidine/putrescine transporter, inner membrane subunit	-1.6
RSP_2099	*murE*	UDP-N-acetylmuramoyl-L-alanyl-D-glutamate--2,6-diaminopimelate ligase	-1.6
RSP_0378	*puuB_2*	Gamma-glutamylputrescine oxidase	-1.6
RSP_6131	.	Putative hydrolase or acyltransferase of alpha/beta superfamily	-1.6
RSP_1877	*ctaD_2*	Cytochrome c oxidase subunit 1	-1.6
RSP_2208	*rbsC_3*	Nucleoside ABC transporter membrane protein	-1.6
RSP_1849	.	Aspartokinase	-1.6
RSP_1361	.	Thioesterase family protein	-1.6
RSP_0017	*potB_1*	ABC spermidine/putrescine transporter, inner membrane subunit	-1.6
RSP_1874	*carA*	Carbamoyl-phosphate synthase small chain	-1.7
RSP_2107	*murG*	DP-N-acetylglucosamine--N-acetylmuramyl-(pentapeptide) pyrophosphoryl-undecaprenol N-acetylglucosamine transferase	-1.7
RSP_1038	*atpB*	ATP synthase subunit a	-1.7
RSP_0002	*hvrA_1*	Histone-like nucleoid-structuring protein H-NS	-1.7
RSP_1066	*engB*	Probable GTP-binding protein EngB	-1.7
RSP_1568	*puuA_3*	Glutamate--putrescine ligase	-1.7
RSP_1580	*lgt*	Phosphatidylglycerol--prolipoprotein diacylglyceryl transferase	-1.7
RSP_6001	.	Peptidase inhibitor I78 family	-1.7
RSP_1290	*livH_4*	Amino acid/amide ABC transporter membrane protein 1, HAAT family	-1.7
RSP_1212	*argG*	Argininosuccinate synthase	-1.7
RSP_2460	*alr*	Alanine racemase	-1.7
RSP_1911	*slt_2*	Putative soluble lytic murein transglycosylase	-1.7
RSP_2108	*murC*	UDP-N-acetylmuramate--L-alanine ligase	-1.7
RSP_0005	*guaA_2*	GMP synthase [glutamine-hydrolyzing]	-1.7
RSP_0964	*sucB*	Dihydrolipoyllysine-residue succinyltransferase component of 2-oxoglutarate dehydrogenase complex	-1.7
RSP_0832	*ychF*	Ribosome-binding ATPase YchF	-1.7
RSP_0251	*bepC*	Outer membrane efflux protein	-1.7
RSP_0092	*sugA_1*	Sorbitol ABC transporter membrane proteinmannitol ABC transporter membrane protein	-1.7
RSP_2693	*sodB*	Superoxide dismutase	-1.7
RSP_0778	*proS*	Proline--tRNA ligase	-1.7
RSP_1484	*cshA*	DNA/RNA helicase, superfamily II	-1.7
RSP_0323	*norB*	Nitric oxide reductase, NorB subunit apoprotein	-1.7
RSP_2781	.	Glutathione-dependent peroxiredoxin	-1.7
RSP_1097	.	Putative zinc protease	-1.7
RSP_6148	.	Putative HPT, Histidine Phosphotransfer domain protein	-1.7

RSP_0155	*echA8_1*	Enoyl-CoA hydratase	-1.7
RSP_1362	.	Major facilitator superfamily (MFS) transporter	-1.7
RSP_1188	*pglF*	Nucleotide sugar epimerase/dehydratase	-1.7
RSP_2110	*murB*	UDP-N-acetylenolpyruvoylglucosamine reductase	-1.7
RSP_2297	*atpA*	ATP synthase subunit alpha	-1.7
RSP_0348	.	ABC transporter, inner membrane subunit	-1.7
RSP_1798	*secD*	Protein translocase subunit SecD	-1.7
RSP_1970	*purN*	Phosphoribosylglycinamide formyltransferase	-1.8
RSP_2950	*cynR*	Transcriptional regulator, LysR family	-1.8
RSP_2812	.	ABC-type uncharacterized transport system, auxiliary component	-1.8
RSP_0707	.	Polyketide cyclase/dehydrase and lipid transport protein	-1.8
RSP_1799	*secF*	Protein translocase subunit SecF	-1.8
RSP_1002	*pyrB*	Aspartate carbamoyltransferase	-1.8
RSP_1017	*blaSE*	Serine protease	-1.8
RSP_0945	*entS*	Major facilitator superfamily (MFS) transporter	-1.8
RSP_2017	.	Uncharacterized protein	-1.8
RSP_1711	*ribN_8*	Putative transporter, RhaT family, DMT superfamily	-1.8
RSP_0372	*argT*	Amino acid ABC transporter substrate-binding protein, PAAT family	-1.8
RSP_2851	*bcr_1*	Multidrug (Bicyclomycin) efflux pump, Major facilitator superfamily (MFS)	-1.8
RSP_1847	*purD*	Phosphoribosylamine--glycine ligase	-1.8
RSP_2355	.	HNH nuclease / Probable phage PHI-105 holin-like protein	-1.8
RSP_0958	.	Transcriptional regulator, AsnC family	-1.8
RSP_0975	.	Succinate dehydrogenase subunit D	-1.8
RSP_1893	*hisN_1*	Inositol monophosphatase family protein	-1.8
RSP_0096	*mtlK*	Mannitol dehydrogenase	-1.8
RSP_0188	*yabI*	Putative membrane-associated protein	-1.9
RSP_1908	.	Outer membrane protein, OmpA/MotB family	-1.9
RSP_2461	*fabG_2*	3-oxoacyl-[acyl-carrier-protein] reductase	-1.9
RSP_2170	*cysP*	Phosphate transporter	-1.9
RSP_0448	*typA*	50S ribosomal subunit assembly factor BipA	-1.9
RSP_2298	*atpG_1*	ATP synthase gamma chain	-1.9
RSP_2221	*purB*	Adenylosuccinate lyase	-1.9
RSP_1941	*cysH*	Phosphoadenylylsulfate reductase (Thioredoxin)	-1.9
RSP_1579	.	Uncharacterized protein	-1.9
RSP_2457	.	Putative paraquat-inducible protein A	-1.9
RSP_1168	.	Parvulin-like PPIase	-1.9
RSP_1036	*atpG_2*	ATP synthase subunit b 2	-1.9
RSP_2100	*murF*	UDP-N-acetylmuramoyl-tripeptide--D-alanyl-D-alanine ligase	-1.9
RSP_1019	.	Putative glycolate oxidase subunit protein	-1.9
RSP_0976	*sdhA*	Succinate dehydrogenase flavoprotein subunit	-1.9

RSP_1514	*ahcY*	Adenosylhomocysteinase	-1.9
RHOS4_29700	.	UPF0246 protein RHOS4_29700	-1.9
RSP_1296	*nagA*	N-acetylglucosamine 6-phosphate deacetylase	-2.0
RSP_2604	*sphX*	Phosphate ABC transporter substrate-binding protein, PhoT family	-2.0
RSP_2550	*exoK*	Endo-beta-1,3-1,4-glycanase protein	-2.0
RSP_0688	*dacF_1*	Putative penicillin-binding protein	-2.0
RSP_1035	*atpF*	ATP synthase subunit b 1	-2.0
RSP_1691	*etfB*	Electron transfer flavoprotein beta subunit	-2.0
RSP_1177	*xylB*	Xylulose kinase	-2.0
RSP_1003	*pyrC_2*	Dihydroorotase-related cyclic amidohydrolase	-2.0
RSP_0900	*camA*	FAD-dependent pyridine nucleotide-disulfide oxidoreductase	-2.0
RSP_0094	*malK_1*	Mannitol ABC transporter ATP-binding proteinsorbitol ABC transporter ATP-binding protein	-2.0
RSP_2459	*mlaE_2*	ABC transporter, inner membrane subunit	-2.0
RSP_1099	*lspA*	Lipoprotein signal peptidase	-2.0
RSP_2455	.	Putative Colicin V production protein, dedE	-2.0
RSP_2243	*hisA*	1-(5-phosphoribosyl)-5-[(5-phosphoribosylamino)methylideneamino] imidazole-4-carboxamide isomerase	-2.0
RSP_0966	*sucD*	Succinate--CoA ligase [ADP-forming] subunit alpha	-2.0
RSP_1404	*pyrF*	Orotidine 5'-phosphate decarboxylase	-2.0
RSP_2198	.	CENP-V/GFA domain-containing protein	-2.0
RSP_2101	*mraY*	Phospho-N-acetylmuramoyl-pentapeptide-transferase	-2.0
RSP_2929	*dsbB*	Putative disulfide bond formation protein DsbB	-2.1
RSP_1037	*atpE*	ATP synthase subunit c	-2.1
RSP_1690	*etfA*	Electron transfer flavoprotein alpha subunit apoprotein	-2.1
RSP_1223	*rutC*	Translation initiation inhibitor, yjgF family / putative Endoribonuclease L-PSP	-2.1
RSP_1100	*purH*	Bifunctional purine biosynthesis protein PurH	-2.1
RSP_2491	*eno*	Enolase	-2.1
RSP_1088	.	Uncharacterized protein	-2.1
RSP_0741	.	Putative metallo-beta-lactamase family protein	-2.1
RSP_1064	*yidC*	Membrane protein insertase YidC	-2.1
RSP_2778	.	Invasion protein B, involved in pathogenesis	-2.1
RSP_1858	*glyS*	Glycine--tRNA ligase beta subunit	-2.1
RSP_2167	.	Putative membrane protein	-2.1
RSP_2539	*cysA_3*	ABC transporter, ATPase subunit	-2.1
RSP_1178	*frcA_1*	Monosaccharide ABC transporter ATP-binding protein, CUT2 family	-2.2
RSP_0859	*leuB*	3-isopropylmalate dehydrogenase	-2.2
RSP_0995	*mnhE1*	Multisubunit potassium/proton antiporter, PhaE subunit	-2.2
RSP_0093	*sugB_1*	Sorbitol ABC transporter membrane proteinmannitol ABC transporter membrane protein	-2.2

RSP_0374	*hisM*	Amino acid ABC transporter membrane protein 2, PAAT family	-2.2
RSP_2199	*dmdC_2*	Putative acyl-CoA dehydrogenase	-2.2
RSP_0669	*tolB*	Tol-Pal system protein TolB	-2.2
RSP_1252	.	2-nitropropane dioxygenase-like protein	-2.2
RSP_1222	.	dITP/XTP pyrophosphatase	-2.3
RSP_1892	.	Putative Cro/CI transcriptional regulator	-2.3
RSP_1940	.	Uncharacterized protein	-2.3
RSP_2868	*guaB_2*	Inosine-5'-monophosphate dehydrogenase	-2.3
RSP_0373	*hisQ*	Amino acid ABC transporter membrane protein 1, PAAT family	-2.3
RSP_0130	*metI*	ABC D-methionine uptake transporter, inner membrane subunit	-2.3
RSP_1289	.	Amino acid/amide ABC transporter membrane protein 2, HAAT family	-2.3
RSP_1179	*xylH*	ABC sugar (Xylose) transporter, inner membrane subunit	-2.3
RSP_0132	*metQ*	ABC D-methionine uptake transporter, substrate-binding protein	-2.3
RSP_0097	*takP*	Alpha-keto acid-binding periplasmic protein TakP	-2.4
RSP_2601	*pstB*	Phosphate import ATP-binding protein PstB	-2.4
RSP_2959	*gapA*	Glyceraldehyde-3-phosphate dehydrogenase	-2.4
RSP_1909	*outD*	Outer membrane general secretion pathway protein, Secretin	-2.4
RSP_1559	*icd_2*	Isocitrate dehydrogenase [NADP]	-2.4
RSP_1176	*xylA*	Xylose isomerase	-2.5
RSP_1354	*bktB*	Beta-ketothiolase	-2.7
RSP_0968	*mdh*	Malate dehydrogenase	-2.7
RSP_0967	*sucC*	Succinate--CoA ligase [ADP-forming] subunit beta	-2.7
RSP_1575	*sat/cysC*	Adenylyl-sulfate kinase	-2.7
RSP_1018	*lutA*	Glycolate oxidase iron-sulfur subunit	-2.7
RSP_1944	*cysG_2*	Uroporphyrinogen III methylase	-2.7
RSP_2602	*pstA2*	Phosphate transport system permease protein PstA	-2.7
RSP_2196	*fadJ*	3-hydroxyacyl-CoA dehydrogenase	-2.8
RSP_2603	*pstC1*	Phosphate transport system permease protein	-2.8
RSP_0910	*dctP*	TRAP-T family transporter, C4-dicarboxylate-binding protein DctP	-2.8
RSP_0993	*mrpC*	Multisubunit potassium/proton antiporter, PhaC subunit	-2.8
RSP_2197	*fadA*	Acetyl-CoA acetyltransferase	-2.9
RSP_1942	*sir*	Sulfite/nitrite reductase hemoprotein subunit	-2.9
RSP_0902	*yieH_1*	Hydrolase, haloacid dehalogenase-like hydrolase	-3.0
RSP_0324	*norC*	Nitric oxide reductase, NorC subunit apoprotein	-3.0
RSP_2172	*metF*	Methylenetetrahydrofolate reductase	-3.1
RSP_1943	.	Uncharacterized protein	-3.3
RSP_6094	*dctM_3*	TRAP-T family sorbitol/mannitol transporter, DctM (12TMs) subunit	-3.3
RSP_0099	.	TRAP-T family sorbitol/mannitol transporter, DctQ (4TMs) subunit	-3.4
RSP_0994	*mrpD*	Multisubunit potassium/proton antiporter, PhaD subunit	-3.5
RSP_1865	*ilvC*	Ketol-acid reductoisomerase (NADP(+))	-3.6

RSP_0911	*dctQ*	TRAP-T family C4-dicarboxylate transporter, DctQ (4TMs) subunit	-3.8
RSP_0912	*dctM_1*	TRAP-T family C4-dicarboxylate transporter, DctM (12TMs) subunit	-3.9
RSP_1615	.	Uncharacterized protein	-4.1
RSP_1613	.	TRAP-T family transporter, DctP subunit	-4.6
RSP_1614	*dctM_5*	TRAP-T family transporter with fused DctQ/DctM subunits	-4.8

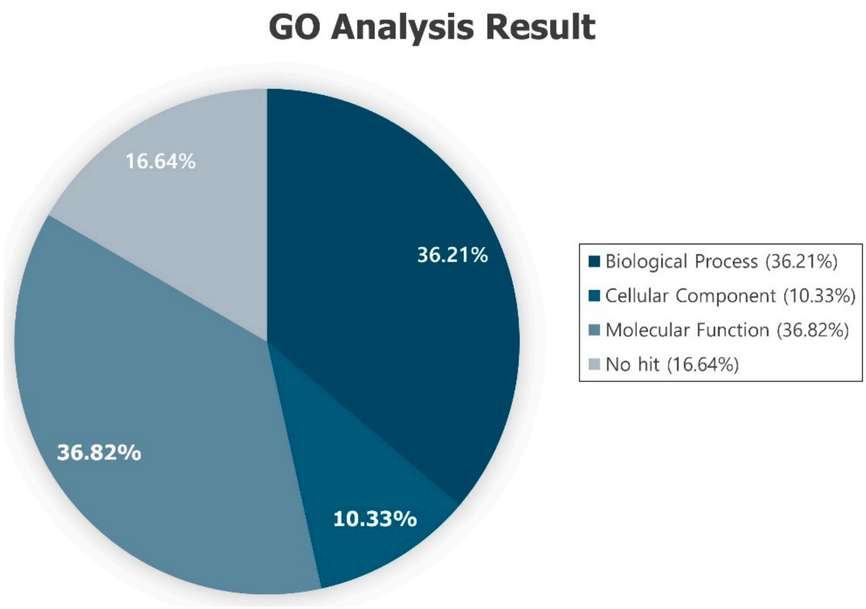

Figure S1. Gene ontology classification of differentially expressed genes. This pie chart depicts the distribution of gene ontology(GO) annotations for genes exhibiting significant differential expression between autotrophic and heterotrophic conditions in *Rhodobacter sphaeroides*. GO provides a standardized nomenclature for gene and gene product functions, categorizing them into three principal domains: Biological Process, Molecular Function, and Cellular Component. Biological Process: Pertains to overarching biological pathways, such as metabolism and signal transduction; the majority of differentially expressed genes were assigned to this domain. Molecular Function: Describes specific biochemical activities, including binding and catalysis; a substantial proportion of genes fell into this category. Cellular Component: Specifies subcellular locales or stable macromolecular complexes associated with gene product localization; fewer genes were annotated here relative to biological process and molecular function. No Hit: Represents the fraction of differentially expressed genes without GO annotations, underscoring potential gaps in current functional annotation knowledge.
